# Can triad forestry reconcile Europe’s biodiversity and forestry strategies? A critical evaluation of forest zoning

**DOI:** 10.1007/s13280-024-02116-2

**Published:** 2024-12-19

**Authors:** Thomas A. Nagel, Mariano Rodríguez-Recio, Tuomas Aakala, Per Angelstam, Admir Avdagić, Zbigniew Borowski, Andrés Bravo-Oviedo, Gediminas Brazaitis, Thomas Campagnaro, Michał Ciach, Milic Curovic, Inken Doerfler, Dimitrios Fotakis, Zoran Govedar, Konstantin Gregor, Yaşar Selman Gültekin, Jacob Heilmann-Clausen, Johanna Hoffmann, Jeňýk Hofmeister, Diāna Jansone, Āris Jansons, Sebastian Kepfer-Rojas, Thibault Lachat, Katharina Lapin, Asko Lõhmus, Michael Manton, Stjepan Mikac, Martin Mikoláš, Frits Mohren, Björn Nordén, Peter Odor, Janine Oettel, Yoan Paillet, Momchil Panayotov, Catalin-Constantin Roibu, Tommaso Sitzia, Miroslav Svoboda, Eszter Tanács, Giovanni Trentanovi, Giorgio Vacchiano, Theo van der Sluis, Tzvetan Zlatanov, Sabina Burrascano

**Affiliations:** 1 Department of Forestry and Renewable Forest Resources, Biotechnical Faculty, University of Ljubljana, Vecna Pot 83, 1000 Ljubljana, Slovenia; 2 Department of Biodiversity, Ecology and Evolution, Faculty of Biological Sciences, Complutense University of Madrid, Madrid, Spain; 3 School of Forest Sciences, University of Eastern Finland, Yliopistokatu 7, 80101 Joensuu, Finland; 4 Department of Forestry and Wildlife Management, Inland Norway University of Applied Sciences, Campus Evenstad, N-2480 Koppang, Norway; 5 Faculty of Forestry, University of Sarajevo, Zagrebačka 20, 71000 Sarajevo, Bosnia and Herzegovina; 6 Department of Forest Ecology, Forest Research Institute, Braci Lesnej 3, Sekocin Stary, 05-090 Raszyn, Poland; 7 Department of Biogeography and Global Change, National Museum of Natural Sciences - CSIC, Serrano 115, 28006 Madrid, Spain; 8 Department of Forest Science, Vytautas Magnus University Agriculture Academy, Studentų 11, 53361 Akademija, Lithuania; 9 Department of Land, Environment, Agriculture and Forestry, Università degli Studi di Padova, 35020 Legnaro, PD Italy; 10 National Biodiversity Future Centre, Piazza Marina, 61, 90133 Palermo, Italy; 11 Department of Forest Biodiversity, Faculty of Forestry, University of Agriculture, al. 29 Listopada 46, 31-425 Kraków, Poland; 12 Biotechnical Faculty, University of Montenegro, Mihaila Lalica 1, 81000 Podgorica, Montenegro; 13 Vegetation Science and Nature Conservation, Institute of Biology and Environmental Science, University of Oldenburg, Oldenburg, Germany; 14 Forest Research Institute, Hellenic Agricultural Organization DIMITRA, 57006 Thessaloniki, Greece; 15 Faculty of Forestry, University of Banja Luka, blvd. Petra Bojovića 1A, 78000 Banja Luka, Bosnia and Herzegovina; 16 TUM School of Life Sciences, Technical University of Munich, Freising, Germany; 17 Forest Economics Department, Faculty of Forestry, Düzce University, Konuralp Yerleskesi, 81620 Düzce, Turkey; 18 Centre for Macroecology, Evolution and Climate, University of Copenhagen, 2100 Copenhagen, Denmark; 19 Austrian Research Centre for Forests, Seckendorff-Gudent-Weg 8, 1131 Vienna, Austria; 20 Department of Forest Ecology, Faculty of Forestry and Wood Sciences, Czech University of Life Sciences Prague, Kamýcká 129, 165 00 Prague, Suchdol, Czech Republic; 21 Latvian State Forest Research Institute “Silava”, Riga Street 111, Salaspils, 2169 Latvia; 22 Department of Geosciences and Natural Resource Mangement, University of Copenhagen, Copenhagen, Denmark; 23 School of Agricultural, Forest and Food Sciences HAFL, Bern University of Applied Sciences, 3052 Zollikofen, Switzerland; 24 WSL Swiss Federal Research Institute, 8903 Birmensdorf, Switzerland; 25 Institute of Ecology and Earth Sciences, University of Tartu, J. Liivi 2, 50409 Tartu, Estonia; 26 Bioeconomy Research Institute, Vytautas Magnus University, Studentų 11, 53361 Akademija, Lithuania; 27 Department of Forest Ecology and Silviculture, Forestry Faculty, University of Zagreb, Zagreb, Croatia; 28 Environmental Sciences Group, Wageningen University & Research, P.O. Box 47, Wageningen, The Netherlands; 29 Norwegian Institute for Nature Research, Sognsveien 68, 0855 Oslo, Norway; 30 Institute of Ecology and Botany, HUN-REN Centre for Ecological Research, Alkotmány u. 2-4, Vácrátót, 2163 Hungary; 31 Institute of Environmental Protection and Nature Conservation, Forestry Faculty, University of Sopron, Bajcsy-Zsilinszky u. 4, Sopron, 9400 Hungary; 32 Université Grenoble Alpes, INRAE, Lessem, 2 rue de la Papeterie, BP76, 38402 Saint Martin d’Heres, France; 33 Dendrology Department, University of Forestry, Kliment Ohridski 10 Blvd., 1757, Sofia, Bulgaria; 34 Forest Biometrics Laboratory, Faculty of Forestry, “Stefan Cel Mare”, University of Suceava, Universitatii Street No. 13, Suceava, Romania; 35 Research Institute On Terrestrial Ecosystems, National Research Council, 50019 Sesto Fiorentino, FI Italy; 36 Department of Agricultural and Environmental Sciences, University of Milan, Milan, Italy; 37 Wageningen Environmental Research, P.O. Box 47, Wageningen, The Netherlands; 38 Institute of Biodiversity and Ecosystem Research, Bulgarian Academy of Sciences, 2 Gagarin Street, 1113 Sofia, Bulgaria; 39 Department of Environmental Biology, Sapienza University of Rome, P.le Aldo Moro 5, 00185 Rome, Italy

**Keywords:** Biodiversity conservation, Disturbance, Forest management, Forest reserve, Land sharing/sparing, Wood production

## Abstract

Balancing increasing demand for wood products while also maintaining forest biodiversity is a paramount challenge. Europe’s Biodiversity and Forest Strategies for 2030 attempt to address this challenge. Together, they call for strict protection of 10% of land area, including all primary and old growth forests, increasing use of ecological forestry, and less reliance on monocultural plantations. Using data on country wide silvicultural practices and a new database on strict forest reserves across Europe, we assess how triad forest zoning could help meet these goals. Our analysis reveals that zoning in Europe is overwhelmingly focused on wood production, while there has been little concomitant protection of forests in strict reserves. Moreover, most strict forest reserves are < 50 ha in size, likely too small to capture the minimum dynamic area necessary to sustain many taxa. We outline research priorities to meet future demands for timber while minimizing the impact on native biodiversity.

**Supplementary Information:**

The online version contains supplementary material available at 10.1007/s13280-024-02116-2.

## Introduction

European forests are vital for human well-being. They are expected to provide habitat for native biodiversity, supply potable water, store and sequester carbon, and meet the growing demand for wood products. Satisfying these various services in the face of a changing climate is a paramount challenge.

Europe’s Biodiversity Strategy for 2030 and Forest Strategy for 2030, the flagship initiatives under the European Green Deal, attempt to address this complex challenge. The Biodiversity Strategy calls for strict protection of 10% of land area, which should include all remaining primary and old growth forests in the European Union. Adhering to the “third of a third rule of thumb” (Hanski 2011), the strategy also calls for conservation and restoration measures on an additional 20% of land, expanding upon Europe’s network of Natura 2000 protected areas, under which forests are often managed with multipurpose forestry that includes timber production. The Forest Strategy calls for increasing the use of integrative forest management that simultaneously fulfils ecological functions and produces timber, namely by using uneven-aged, continuous cover forestry with diverse tree species mixtures. The Forest Strategy suggests that such forests should be promoted instead of high-yield monocultural plantations, and that clear-cutting should be avoided.

However, placing additional forest area under strict protection and expanding forests managed with extensive, integrated management instead of high-yield plantations may lead to a decline in future timber production in Europe. Current trajectories project an increase in timber demand both worldwide and in Europe, with global roundwood consumption expected to increase by 54% by 2050 (Peng et al. 2023). The new EU Strategies offer little to reconcile enlarging and improving forest habitat for native biodiversity with the increasing demand for timber.

Aside from a goal of strictly protecting 10% of land area, both strategies are largely based on the concept of land sharing, whereby forests are managed to simultaneously fulfil ecological and timber production goals. However, there is little science-based evidence that a sharing approach is the best strategy to maintain or increase timber production at least cost to biodiversity over large areas. In fact, some European studies indicate that widespread sharing in forestry leads to regional declines in biodiversity that is dependent on old growth forest conditions, particularly species dependent on large amounts of deadwood, habitat trees, and disturbance legacies (Gossner et al. 2013; Nagel et al. 2017a).

The alternative to a land sharing approach is to use high-yield timber plantations to satisfy timber demand, while retaining other parts of the forested landscape in unmanaged, strictly protected reserves for the maintenance of native biodiversity, otherwise referred to as land sparing. There are numerous zoning solutions between the extreme sharing and sparing that represent opposite ends of a continuum. For example, a well-known three compartment approach in forest planning includes the triad management first proposed by Seymour and Hunter (1992). Triad includes areas managed with high-yield plantations (Paquette and Messier 2010), unmanaged areas that protect or allow the development of old growth or primary forests, and areas managed with lower-yielding, extensive management, forming the forest matrix between the other zones (Franklin and Lindenmayer 2009). Among the few studies that examine how forest zoning approaches influence trade-offs between timber production and biodiversity, sparing approaches and triad often outperform sharing (Ranius and Roberge 2011; Trivino et al. 2017; Blattert et al. 2023; Harris and Betts 2023). However, there is still a scarcity of research providing guidance on the optimal proportions of land under triad compartments under different levels of timber demand, or the spatial scale and arrangement of these compartments across different regions (Betts et al. 2021).

Using data on country wide silvicultural practices and a newly compiled database on strict forest reserves across Europe, we assess the zoning of forest functions using triad as a framework. Our analysis reveals that current zoning in Europe is overwhelmingly focused on wood production, while there has been little concomitant protection of forests in strict reserves to balance this production focus. Moreover, based on knowledge of natural disturbance regimes, most strict forest reserves in Europe are likely too small to capture the minimum dynamic area that would sustain habitats for both old growth and disturbance dependent taxa. We discuss these findings in the context of European forest-related policy, and outline future research priorities aimed at establishing a science-based pathway to meet future demands for timber while ensuring conservation of viable populations of native biodiversity.

## Triad assessment

Our assessment of triad zoning in Europe is based on the area of forests under intensive management, extensive management, and strict protection. Data on strictly protected forests were compiled for this analysis from the most recent information available in each country, and includes the total area and size distribution of forests under strict protection in 27 European countries (Appendix S1). Strict forest reserves were defined as areas where forests develop under natural processes, such that any type of wood extraction is prohibited, including sanitation or salvage logging after disturbance. In general, strict forest reserves for most countries are part of a national or regional network of forest reserves, or consist of core areas of national parks. Additionally, we required a minimum size of 5 ha to separate very small protected patches and habitat features within extensively managed forests (e.g. land sharing approach) from forest reserves (e.g. sparing). In cases where a large protected area included other ecosystem types (e.g. alpine grassland or other non-forest ecosystems), we only included the share of forest area. Finally, all areas included in the database were required to be protected under a legal framework, such as under national or regional regulations. Therefore, unmanaged forests lacking formal protection status were not included.

Data on the proportion of intensive and extensive forest management across the same countries were extracted from the study conducted by Mason et al. (2021), which contains up-to-date information on the proportion of forests managed with different silvicultural systems. Their assessment included data on silvicultural systems under both even-aged, rotational forest management (e.g. clear-felling, uniform shelterwood, and seed tree systems) and uneven-aged, continuous cover management (e.g. single tree selection, group selection, irregular shelterwood). For our assessment, we aggregated the data from even-aged systems to represent intensive management, and the data from uneven-aged systems to represent extensive management. We acknowledge that this simple classification has drawbacks. For example, forests managed with uneven-aged systems that focus on timber production can have small target diameters and very little deadwood, while some even-aged systems can retain large trees and high amounts of deadwood. Moreover, some may consider even-aged shelterwood or small clearcuts as a type of extensive management. However, the treatment size and rotation period applied in Europe places these systems far outside the natural disturbance regime (Aszalós et al. 2022). Finally, our assessment makes a simple assumption that forest land that is not protected in strict forest reserves is available for management, consistent with data suggesting that about 85% of European forest area is available for wood supply (Forest Europe 2020).

**Fig. 1:**
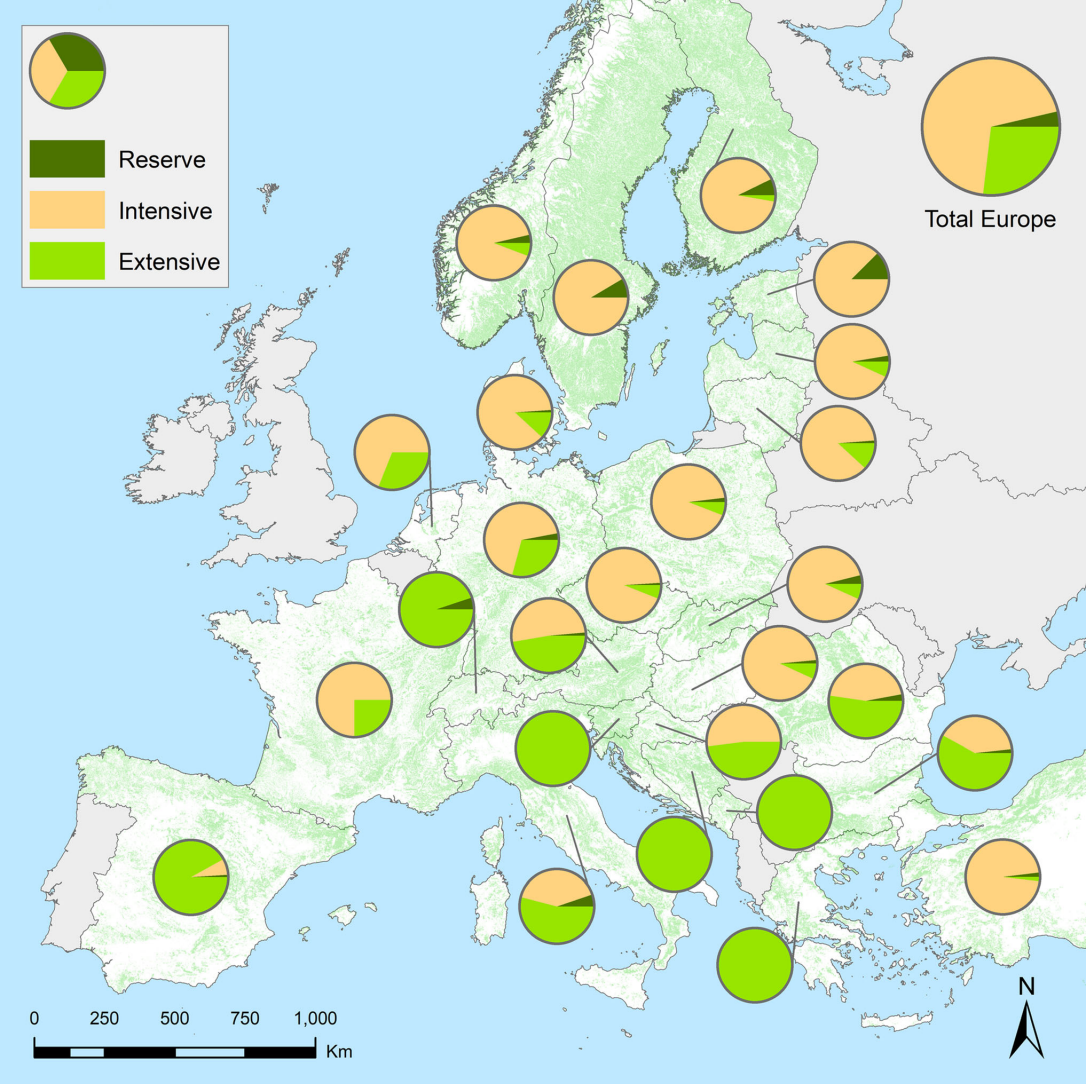
Country level triad zoning across Europe, with pie charts showing the proportion of strictly protected forest reserves (i.e. no timber harvesting), intensively managed forests (i.e. even-aged, rotational management), and extensively managed forests (i.e. uneven-aged, continuous cover management) out of the total forest area in each country (green background on map). Total forest area in each country was extracted from 2020 country reports from the FAO Global Forest Resources Assessment. Note that zones representing less than 1% of total forest area are not shown on pie charts

Within any given country, the data show that current forest zoning across Europe substantially diverges from a triad system, assuming a balanced division among the three triad zones for the sake of discussion (Fig. 1). Countries in the south-eastern part of the temperate zone (e.g. Slovenia, Bosnia and Herzegovina, Montenegro) mostly use extensive management, and allocate less than 1% of their forest area to strict protection. Most countries in Central and Northern Europe prioritize intensive timber production, with relatively little area devoted to either extensive management or strict protection. Only one country (Italy) partly resembles a triad system at the national level. One country has > 10% of forest area under strict protection (Estonia: 12%), and several other countries are approaching 10% (Sweden: 9%; Finland: 7%), but most countries have set aside less than 2% of forest area for strict protection. As a whole, 3.6% of the total forest area in the dataset, representing most of Europe, is under strict protection, which indicates an upward trend since the 1999, when approximately 1.7% of European forests were strictly protected (Parviainen et al. 2000).

## Strict reserve size and natural disturbance regimes

**Fig. 2:**
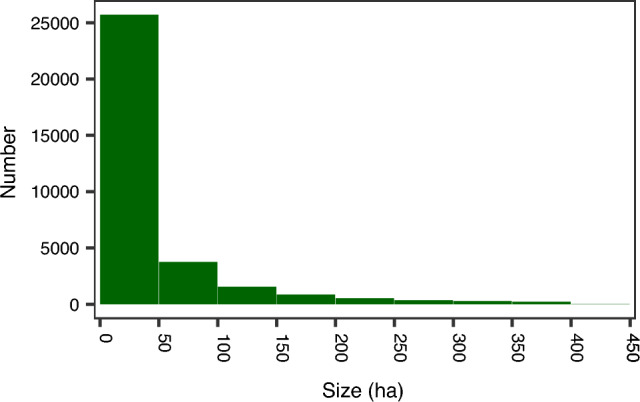
Size distribution of strict forest reserves in Europe, showing the portion of the dataset below the 95^th^ percentile. Note that the data include the total size of individual reserves that are completely forested, or the area of forests within strict reserves that also include non-forest ecosystems

Among the 35 080 strictly protected forest areas in the dataset, 73% are under 50 ha in size and 53% are under 20 ha in size (Fig. 2). While there are some exceptionally large areas (i.e. > 100 000 ha in Finland and Sweden), only 2% are > 1000 ha and 52 areas are > 10 000 ha, most of which are found in Sweden (*n* = 21), Finland (*n* = 17), and Turkey (*n* = 7). Large reserves also make up a disproportionate amount of the total area under strict protection. For example, reserves > 1000 ha in size collectively make up 46 867 km^2^ (70% of total area under strict protection), compared to 4009 km^2^ (6% of total) for reserves under 50 ha.

This widespread lack of large, strictly protected forested landscapes, where old growth and early seral conditions can develop under regimes of natural disturbances, is cause for concern with regard to biodiversity conservation. Among other reasons, the dominance of small forest reserves may be due to a traditional, but outdated understanding of forest dynamics, in which forest development is thought to be regulated by continuous, diffuse mortality of single or small groups of old trees (e.g. gap dynamics), giving rise to a relatively steady-state forest structure at small scales (e.g. < 50 ha). However, a rich history of disturbance ecology research in Europe during the past few decades clearly demonstrates that disturbances are an integral part of forest dynamics (Kulakowski et al. 2017). Natural disturbances, such as windstorms, ice-storms, wildfires, and bark beetle outbreaks, periodically interrupt the ongoing process of gap dynamics and give rise to heterogeneous mortality patterns in forested landscapes, ranging from small patches of canopy removal to entire stands or landscapes capturing a range of damage severities. The legacies created by these disturbances, including standing, snapped, and uprooted trees, large inputs of sun-exposed deadwood, and early seral vegetation, serve as key habitat for many taxa, yet many of these legacies are often routinely removed during forest management (Thorn et al. 2020).

**Fig. 3:**
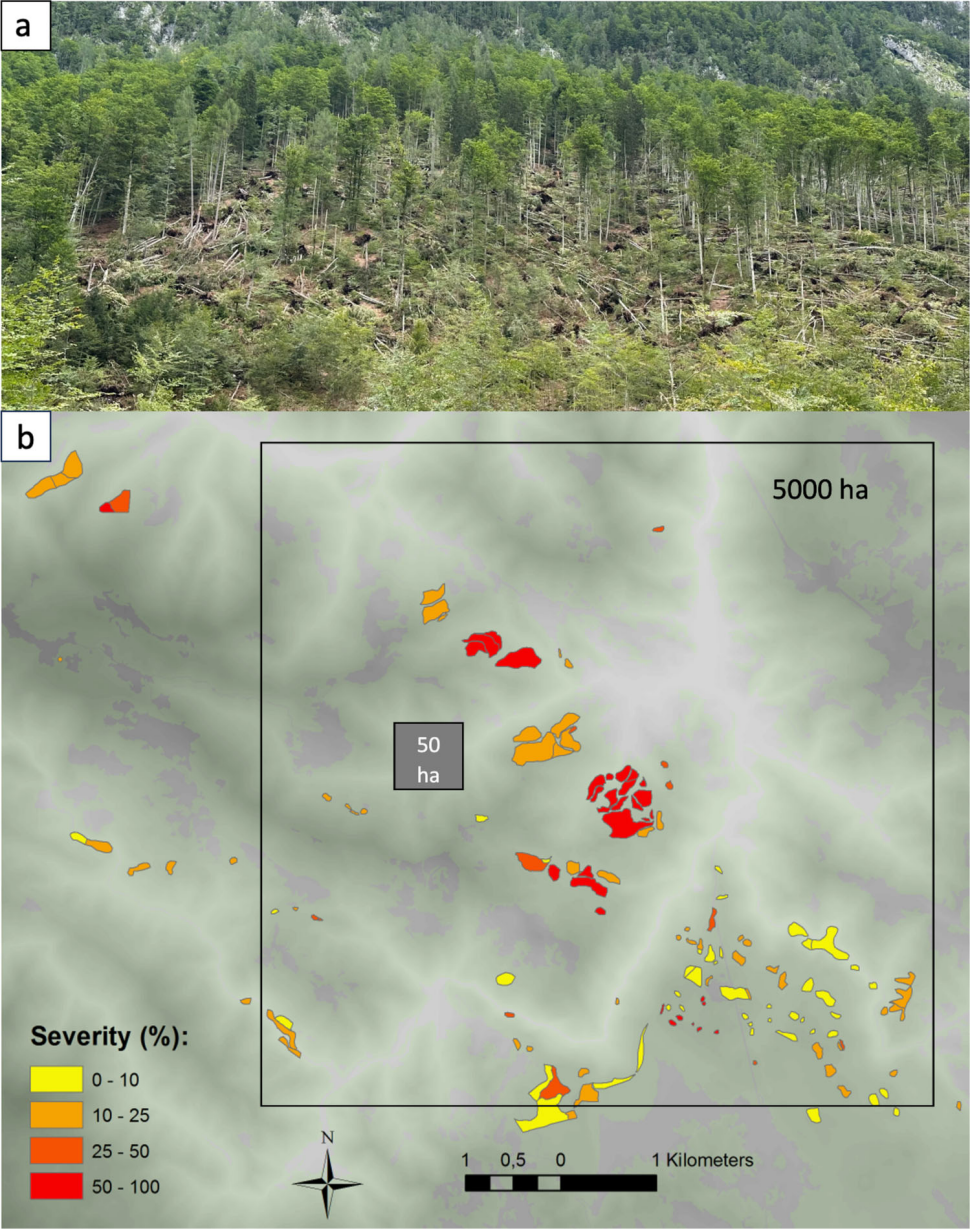
**a** Wind disturbance damage caused by a summer thunderstorm in a temperate *Fagus sylvatica* dominated forest in Europe, showing typical patch-scale partial canopy removal and disturbance legacies, such as abundant sun-exposed deadwood, tip and mound habitat, and windfirm legacy trees. **b** Landscape-scale distribution of disturbance patches of varying size and severity from a summer thunderstorm in temperate forests of Slovenia (from Nagel et al. 2017b). The box depicts the size of a large reserve (e.g. 5000 ha) needed to capture such events compared to the typical small reserve (e.g. 50 ha)

Capturing the natural disturbance regime requires large protected areas, sometimes referred to as a *minimum dynamic area*. Pickett and Thompson (1978) defined this as “the smallest area with a natural disturbance regime which maintains internal recolonization sources and hence minimizes extinctions”. To provide an example from temperate mountain forests of Europe, intermediate severity disturbance events, such as blowdown patches from convective storms, are an important component of the natural disturbance regime. These events tend to cause stand-scale damage to forests (e.g. 10s of ha) with heterogeneous severity patterns, ranging from small gaps to larger blowdown patches varying in damage severity (Nagel et al. 2017b) (Fig. 3). Such events have return intervals of several centuries (Nagel et al. 2014), which implies that large landscapes are required to encompass a mosaic of stands recovering from past disturbances (Fig.  3). For example, research in boreal forest ecosystems suggest minimum sizes of > 5000 ha (Edwards et al. 2022).

## Research and policy recommendations

Very little forest area is strictly protected in Europe, even in regions that devote most of their forests to intensive wood production, where one might expect there could be scope for larger areas under strict protection to balance timber production. Many native species can thrive in forests outside of strictly protected areas in Europe (Chapron et al. 2014; Schall et al. 2018), implying that a sharing approach can fulfil both timber production and conservation of much native biodiversity. However, there is also a substantial body of research demonstrating that many forest dwelling species are tightly connected with conditions found in old growth and primary forests, especially species dependent on old habitat trees, decaying deadwood, and disturbance legacies associated with early seral conditions (e.g. saproxylic species of lichens, bryophytes, fungi, insects, birds, and bats) (e.g. Wesołowski 2005; Brunet et al. 2010; Nagel et al. 2017a; Eckelt et al. 2018; Thorn et al. 2020; Kozák et al. 2021; Mikolas et al. 2021; Gloor et al. 2024). The latter body of research calls for protecting existing old growth and primary forests, increasing the area of strictly protected forests, and improving habitat conditions in extensively managed forests. Triad forestry would seemingly offer a viable solution to reconcile the increasing demand for timber, conservation of native biodiversity, and the development of closer-to-nature forestry practices (Larsen et al. 2022). However, few countries employ a regional triad system, and there are many key questions that require evidence-based answers before triad systems can be implemented. Below we highlight several research and policy priorities to this end:

1. The strict protection of 10% of land area in Europe called for under the EU Biodiversity Strategy should include a sufficient amount of forest area. For example, 10% of total forest area may be a conservative target within most regions, particularly given that forests are the natural late-successional vegetation cover in the absence of management across most of Europe, yet cover less than half of the continent. We also reiterate the call for rapid protection of remaining primary and old-growth forests (Mikolas et al. 2023).
2. In addition to the many small strict forest reserves in Europe that are important for protecting key habitats and species (i.e. fine filter approach), larger reserves that capture natural disturbance regimes are also needed (i.e. coarse filter). As countries seek to expand land area under strict protection in Europe, there should be an emphasis on including some large forested landscapes whenever feasible. Further research is also needed to quantify the minimum dynamic area for different forest types in Europe, which requires data on disturbance regime components, such as patch size and frequency. In this regard, remote sensing of forest disturbances over extant primary forest landscapes could provide valuable reference conditions. Likewise, further research on the area requirements of species associated with old growth is needed. Recent work, for example, indicates that the White-backed Woodpecker, a rare deadwood dependent species restricted to broadleaf forests with old growth structures in Europe, requires habitat patches of about 300 ha (Campion et al. 2020). The White-backed Woodpecker is also a known umbrella species for other forest biodiversity, including bird species of conservation concern and threatened saproxylic beetles (Roberge et al. 2008; Angeleri et al. 2024), and may thus serve as an effective indicator for identifying and protecting new strict forests reserves.
3. Policies aimed at improving and expanding extensive management, such as forests managed with closer-to-nature principles, should follow ecological forestry guidelines based on studies of natural disturbance regimes and target values for retaining key forest structures (e.g. minimum habitat tree density and deadwood volume) (Larsen et al. 2022; Kuuluvainen and Pukkala 2024; Nagel et al. 2024). We acknowledge that our exclusion of even-aged systems from the extensive management zone is not necessarily consistent with ecological forestry, as there could be cases when even-aged stands are emulative of natural disturbance dynamics (Kaasik et al. 2023). Continued research is needed to examine how ecological forestry influences both timber production and biodiversity, especially with regard to forest structural requirements for taxa dependent on old growth conditions.
4. Research is needed to quantify the optimal proportions, scale, and spatial configuration of land area under triad compartments across different social-ecological systems in different ecoregions/countries in Europe, with the goal of meeting rising demand for wood production while maintaining native forest biodiversity. The implementation and challenges associated with land sharing-sparing research in forests are well documented by Betts et al. (2021), and will likely require long-term empirical experiments, observational studies, and simulation models. There are some research directions that can be quickly pursued, such as leveraging existing databases that include both data on multi-taxa biodiversity and forest management history (Burrascano et al. 2023), or using dynamic vegetation models that can simulate forest yield and biodiversity habitat across virtual triad treatments. In a recent example using a dynamic vegetation model and optimization of multiple ecosystem services (e.g. carbon storage, biodiversity habitat, and wood production), Gregor et al. (2022) identified an optimized Europe-wide portfolio that contains 29% unmanaged forests, mainly due to the co-benefit between carbon storage and biodiversity habitat provided in unmanaged forests. In a follow up study, introducing a constraint of 10% strict forest protection and stable timber harvest levels, Gregor et al. (2024) identified substantial trade-offs in the provision of ecosystem services and timber production across Europe, whereby some regions would need to prioritize timber production to make up for reduced harvests elsewhere. They call for additional research using regional optimizations based on higher resolution data, existing old growth and primary forests areas, ownership structure, and forest accessibility to better address these conflicting demands and coordinate solutions across Europe (Gregor et al. 2024).
5. Europe has a large proportion of non-industrial private forest ownership, often fragmented into small land holdings, many of which are not regularly managed for wood production. This ownership patchwork creates both challenges and opportunities for the implementation of large-scale triad treatments, and the optimal configuration of potential triad treatments will likely vary across regions with different patterns of forest ownership (Naumov et al. 2018). Research will therefore need to incorporate ownership patterns and data on private forest management into models. Policies should focus on creating new incentives to encourage owners to regularly manage forests and contribute to wood production in Europe, or to designate forests with old growth features as strict reserves.

## Conclusions

Implementing the new Biodiversity and Forest Strategies will be challenging given predictions of increasing demand for timber and the increasing threat of climate change. Triad may offer a valuable framework for meeting these growing demands for domestic timber production, while still maintaining sufficient and well-connected habitat for forest biodiversity, including those species that require large unmanaged forest landscapes. We also emphasize that triad zoning could potentially accommodate other forest functions, including climate change adaptation and mitigation strategies. For example, recent work demonstrates the high carbon carrying capacity in old growth and primary forests across Europe, and the mitigation potential if additional forests are protected in strict reserves (Keith et al. 2024). As a compliment to strict reserves, zones focused on timber production have high adaptation potential, as forest managers can adjust silvicultural regimes and tree species composition toward species adapted to future conditions (Pawson et al. 2013). Although implementing triad zoning in Europe presents challenges, it is not an insurmountable task. We hope that this paper will serve as a catalyst for expanded research on the efficacy of triad zoning and stimulate discussion on how to effectively achieve the goals outlined in the new European strategies related to forestry and biodiversity.

## Supplementary Information

Below is the link to the electronic supplementary material.

Supplementary file1 (PDF 4869 KB)

## Data Availability

The dataset on strict forest reserves used in this study is available at https://zenodo.org/records/14228225.

## References

[CR1] Angeleri, R., U.G. Kormann, N. Roth, A. Ettwein, G. Pasinelli, R. Arlettaz, and T. Lachat. 2024. The White-backed Woodpecker (*Dendrocopos leucotos*) as an umbrella species for threatened sraproxylic beetle communities in Central European beech forests. *Ecological Indicators* 167: 112632. 10.1016/j.ecolind.2024.112632.

[CR2] Aszalós, R., D. Thom, T. Aakala, P. Angelstam, G. Brūmelis, L. Gálhidy, G. Gratzer, T. Hlásny, et al. 2022. Natural disturbance regimes as a guide for sustainable forest management in Europe. *Ecological Applications* 32: e2596. 10.1002/eap.2596. 35340078 10.1002/eap.2596

[CR3] Betts, M.G., B.T. Phalan, C. Wolf, S.C. Baker, C. Messier, K.J. Puettmann, R. Green, S.H. Harris, et al. 2021. Producing wood at least cost to biodiversity: Integrating triad and sharing-sparing approaches to inform forest landscape management. *Biological Reviews* 96: 1301–1317. 10.1111/brv.12703. 33663020 10.1111/brv.12703

[CR4] Blattert, C., K. Eyvindson, M. Monkkonen, K.J. Raatikainen, M. Trivino, and R. Duflot. 2023. Enhancing multifunctionality in European boreal forests: The potential role of Triad landscape functional zoning. *Journal of Environmental Management* 348: 119250. 10.1016/j.jenvman.2023.119250. 37864945 10.1016/j.jenvman.2023.119250

[CR5] Brunet, J., O. Fritz, and G. Richnau. 2010. Biodiversity in European beech forests-a review with recommendations for sustainable forest management. *Ecological Bulletins* 53: 77–94.

[CR6] Burrascano, S., F. Chianucci, G. Trentanovi, S. Kepfer-Rojas, T. Sitzia, F. Tinya, I. Doerfler, Y. Pailletj, et al. 2023. Where are we now with European forest multi-taxon biodiversity and where can we head to? *Biological Conservation* 284: 110176. 10.1016/j.biocon.2023.110176.

[CR7] Campion, D., I. Pardo, M. Elosegui, and D. Villanua. 2020. GPS telemetry and home range of the White-backed Woodpecker *Dendrocopos leucotos*: Results of the first experience. *Acta Ornithologica* 55: 77–87. 10.3161/00016454AO2020.55.1.008.

[CR8] Chapron, G., P. Kaczensky, J.D.C. Linnell, M. von Arx, D. Huber, H. Andren, J. Vicente Lopez-Bao, M. Adamec, et al. 2014. Recovery of large carnivores in Europe’s modern human-dominated landscapes. *Science* 346: 1517–1519. 10.1126/science.1257553. 25525247 10.1126/science.1257553

[CR9] Eckelt, A., J. Müller, U. Bense, H. Brustel, H. Bußler, Y. Chittaro, L. Cizek, A. Frei, et al. 2018. “Primeval forest relict beetles” of Central Europe: A set of 168 umbrella species for the protection of primeval forest remnants. *Journal of Insect Conservation* 22: 15–28. 10.1007/s10841-017-0028-6.

[CR10] Edwards, M., K. Lisgo, S. Leroux, M. Krawchuk, S. Cumming, and F. Schmiegelow. 2022. Conservation planning integrating natural disturbances: Estimating minimum reserve sizes for an insect disturbance in the boreal forest of eastern Canada. *PLoS ONE* 17: e0268236. 10.1371/journal.pone.0268236. 35533149 10.1371/journal.pone.0268236 PMC9084528

[CR11] Forest Europe. 2020. State of Europe’s Forests 2020.

[CR12] Franklin, J.F., and D.B. Lindenmayer. 2009. Importance of matrix habitats in maintaining biological diversity. *Proceedings of the National Academy of Sciences of the United States of America* 106: 349–350. 10.1073/pnas.0812016105. 19129497 10.1073/pnas.0812016105 PMC2626705

[CR13] Gloor, R., M. Svitok, M. Mikoláš, J. Hofmeister, J. Halda, P. Janda, F.M. Sabatini, L. Zemanová, et al. 2024. Sustaining forest biodiversity: Exploring the effect of long-term natural disturbance dynamics on contemporary lichen communities in primary forest ecosystems. *Forest Ecosystems* 11: 100214. 10.1016/j.fecs.2024.100214.

[CR14] Gossner, M.M., T. Lachat, J. Brunet, G. Isacsson, C. Bouget, H. Brustel, R. Brandl, W.W. Weisser, et al. 2013. Current near-to-nature forest management effects on functional trait composition of saproxylic beetles in beech forests: functional diversity of beetles. *Conservation Biology* 27: 605–614. 10.1111/cobi.12023. 23432190 10.1111/cobi.12023

[CR15] Gregor, K., T. Knoke, A. Krause, C.P.O. Reyer, M. Lindeskog, P. Papastefanou, B. Smith, A.-S. Lanso, et al. 2022. Trade-offs for climate-smart forestry in Europe under uncertain future climate. *Earths Future* 10: e2022EF002796. 10.1029/2022EF002796.

[CR16] Gregor, K., C.P.O. Reyer, T.A. Nagel, A. Mäkelä, A. Krause, T. Knoke, and A. Rammig. 2024. Reconciling the EU forest, biodiversity, and climate strategies. *Global Change Biology* 30: e17431. 10.1111/gcb.17431. 39092769 10.1111/gcb.17431

[CR17] Hanski, I. 2011. Habitat loss, the dynamics of biodiversity, and a perspective on conservation. *Ambio* 40: 248–255. 10.1007/s13280-011-0147-3. 21644453 10.1007/s13280-011-0147-3 PMC3357798

[CR18] Harris, S.H., and M.G. Betts. 2023. Selecting among land sparing, sharing and Triad in a temperate rainforest depends on biodiversity and timber production targets. *Journal of Applied Ecology* 60: 737–750. 10.1111/1365-2664.14385.

[CR19] Kaasik, A., R. Kont, and A. Lohmus. 2023. Modeling forest landscape futures: Full scale simulation of realistic socioeconomic scenarios in Estonia. *PLoS ONE* 18: e0294650. 10.1371/journal.pone.0294650. 37976263 10.1371/journal.pone.0294650 PMC10655990

[CR20] Keith, H., Z. Kun, S. Hugh, M. Svoboda, M. Mikolas, D. Adam, D. Bernatski, V. Blujdea, et al. 2024. Carbon carrying capacity in primary forests shows potential for mitigation achieving the European Green Deal 2030 target. *Communications Earth & Environment* 5: 256. 10.1038/s43247-024-01416-5.

[CR21] Kozák, D., M. Svitok, M. Wiezik, M. Mikoláš, S. Thorn, A. Buechling, J. Hofmeister, R. Matula, et al. 2021. Historical disturbances determine current taxonomic, functional and phylogenetic diversity of saproxylic beetle communities in temperate primary forests. *Ecosystems* 24: 37–55. 10.1007/s10021-020-00502-x.

[CR22] Kulakowski, D., R. Seidl, J. Holeksa, T. Kuuluvainen, T.A. Nagel, M. Panayotov, M. Svoboda, S. Thorn, et al. 2017. A walk on the wild side: Disturbance dynamics and the conservation and management of European mountain forest ecosystems. *Forest Ecology and Management* 388: 120–131. 10.1016/j.foreco.2016.07.037. 28860677 10.1016/j.foreco.2016.07.037 PMC5572638

[CR23] Kuuluvainen, T., and T. Pukkala. 2024. Ecological silviculture for fennoscandian scots pine ecosystems. In *Ecological silvicultural systems: Exemplary models for sustainable forest management*, ed. B.J. Palik and A.W. D’Amato, 229–243. Hoboken, NJ: Wiley.

[CR24] Larsen, J. B., P. Angelstam, J. Bauhus, J. F. Carvalho, J. Diaci, D. Dobrowolska, A. Gazda, L. Gustafsson, et al. 2022. *Closer-to-Nature Forest Management. From Science to Policy 12.* Vol. 12. EFI European Forest Institute.

[CR25] Mason, W.L., J. Diaci, J. Carvalho, and S. Valkonen. 2021. Continuous cover forestry in Europe: Usage and the knowledge gaps and challenges to wider adoption. *Forestry: an International Journal of Forest Research* 95: 1–12. 10.1093/forestry/cpab038.

[CR26] Mikolas, M., M. Svitok, R. Bace, G.W. Meigs, W.S. Keeton, H. Keith, A. Buechling, V. Trotsiuk, et al. 2021. Natural disturbance impacts on trade-offs and co-benefits of forest biodiversity and carbon. *Proceedings of the Royal Society B-Biological Sciences* 288: 20211631. 10.1098/rspb.2021.1631. 10.1098/rspb.2021.1631 PMC8527197 34666524

[CR27] Mikolas, M., G. Piovesan, A. Ahlstrom, D.C. Donato, R. Gloor, J. Hofmeister, W.S. Keeton, B. Muys, et al. 2023. Protect old-growth forests in Europe now. *Science* 380: 466. 10.1126/science.adh2303. 10.1126/science.adh2303 37141361

[CR28] Nagel, T.A., M. Svoboda, and M. Kobal. 2014. Disturbance, life history traits, and dynamics in an old-growth forest landscape of southeastern Europe. *Ecological Applications* 24: 663–679. 24988767 10.1890/13-0632.1

[CR29] Nagel, T.A., D. Firm, R. Pisek, T. Mihelic, D. Hladnik, M. de Groot, and D. Rozenbergar. 2017a. Evaluating the influence of integrative forest management on old-growth habitat structures in a temperate forest region. *Biological Conservation* 216: 101–107. 10.1016/j.biocon.2017.10.008.

[CR30] Nagel, T.A., S. Mikac, M. Dolinar, M. Klopcic, S. Keren, M. Svoboda, J. Diaci, A. Boncina, et al. 2017b. The natural disturbance regime in forests of the Dinaric mountains: A synthesis of evidence. *Forest Ecology and Management* 388: 29–42. 10.1016/j.foreco.2016.07.047.

[CR31] Nagel, T.A., M. Svoboda, L. Vitkova, and D. Rozenbergar. 2024. Ecological silviculture for european beech-dominated forest ecosystems. In *Ecological silvicultural systems: exemplary models for sustainable forest management*, ed. B.J. Palik and A.W. D’Amato, 257–270. Hoboken, NJ: Wiley.

[CR32] Naumov, V., M. Manton, M. Elbakidze, Z. Rendenieks, J. Priednieks, S. Uhlianets, T. Yamelynets, A. Zhivotov, et al. 2018. How to reconcile wood production and biodiversity conservation? The Pan-European boreal forest history gradient as an “experiment.” *Journal of Environmental Management* 218: 1–13. 10.1016/j.jenvman.2018.03.095. 29660541 10.1016/j.jenvman.2018.03.095

[CR33] Paquette, A., and C. Messier. 2010. The role of plantations in managing the world’s forests in the Anthropocene. *Frontiers in Ecology and the Environment* 8: 27–34. 10.1890/080116.

[CR34] Parviainen, J., W. Bücking, K. Vandekerkhove, A. Schuck, and R. Päivinen. 2000. Strict forest reserves in Europe: Efforts to enhance biodiversity and research on forests left for free development in Europe (EU-COST-Action E4). *Forestry* 73: 107–118. 10.1093/forestry/73.2.107.

[CR35] Pawson, S.M., A. Brin, E.G. Brockerhoff, D. Lamb, T.W. Payn, A. Paquette, and J.A. Parrotta. 2013. Plantation forests, climate change and biodiversity. *Biodiversity and Conservation* 22: 1203–1227. 10.1007/s10531-013-0458-8.

[CR36] Peng, L., T.D.D. Searchinger, J. Zionts, and R. Waite. 2023. The carbon costs of global wood harvests. *Nature* 620: 110. 10.1038/s41586-023-06187-1. 37407827 10.1038/s41586-023-06187-1 PMC10396961

[CR37] Pickett, S., and J. Thompson. 1978. Patch dynamics and design of nature reserves. *Biological Conservation* 13: 27–37. 10.1016/0006-3207(78)90016-2.

[CR38] Ranius, T., and J.-M. Roberge. 2011. Effects of intensified forestry on the landscape-scale extinction risk of dead wood dependent species. *Biodiversity and Conservation* 20: 2867–2882. 10.1007/s10531-011-0143-8.

[CR39] Roberge, J.-M., G. Mikusinski, and S. Svensson. 2008. The white-backed woodpecker: Umbrella species for forest conservation planning? *Biodiversity and Conservation* 17: 2479–2494. 10.1007/s10531-008-9394-4.

[CR40] Schall, P., M.M. Gossner, S. Heinrichs, M. Fischer, S. Boch, D. Prati, K. Jung, V. Baumgartner, et al. 2018. The impact of even-aged and uneven-aged forest management on regional biodiversity of multiple taxa in European beech forests. *Journal of Applied Ecology* 55: 267–278. 10.1111/1365-2664.12950.

[CR41] Seymour, R. S., and M. L. Hunter. 1992. *New Forestry in Eastern Spruce-Fir Forests: Principles and Applications to Maine*. 716. Maine Agricultural and Forest Experiment Station Miscellaneous Publication.

[CR42] Thorn, S., S. Seibold, A.B. Leverkus, T. Michler, J. Mueller, R.F. Noss, N. Stork, S. Vogel, et al. 2020. The living dead: acknowledging life after tree death to stop forest degradation. *Frontiers in Ecology and the Environment* 18: 505–512. 10.1002/fee.2252.

[CR43] Trivino, M., T. Pohjanmies, A. Mazziotta, A. Juutinen, D. Podkopaev, E. Le Tortorec, and M. Monkkonen. 2017. Optimizing management to enhance multifunctionality in a boreal forest landscape. *Journal of Applied Ecology* 54: 61–70. 10.1111/1365-2664.12790.

[CR44] Wesołowski, T. 2005. Virtual conservation: how the European Union is turning a blind eye to its vanishing primeval forests. *Conservation Biology* 19: 1349–1358. 10.1111/j.1523-1739.2005.00265.x.

