# Supplementary material for: Can triad forestry reconcile Europe’s biodiversity and forestry strategies? A critical evaluation of forest zoning
Source: Ambio. 2024 Dec 19;54(4):632–41. doi: 10.1007/s13280-024-02116-2 (PMC11871248; doi:10.1007/s13280-024-02116-2)
Supplement: Supplementary file 1 — Supplementary file1 (PDF 4869 KB) [file 13280_2024_2116_MOESM1_ESM.pdf]

Ambio

Supplementary Information

Title: Can triad forestry reconcile Europe's biodiversity and forestry strategies? A critical evaluation of forest zoning

Appendix S1. Description of strict forest reserves for countries included in the dataset, including important methodological considerations.

Protected area networks within European countries are complex, often characterized by an array of protection categories and with multiple management agencies within any given country. These various forms of protection sometimes have definitions that are difficult to interpret, resulting in considerable confusion with regard to the type of management activities that are allowed in a given protected area. This is especially problematic when trying to identify strict forest reserves (i.e. protected areas in forest ecosystems where timber extraction and salvage/sanitation logging are not allowed, and where forest dynamics are regulated by natural processes). The complexities and general lack of harmonisation among protected forest categories across Europe have been highlighted several times in the past (Frank et al. 2007; Parviainen et al. 2000). However, these problems still persist today and create a major obstacle to fulfil European policies that call for increased protected areas, including strict protection of forests.

Given these challenges, we acknowledge that the dataset on strictly protected forests compiled for this study is likely not complete. Moreover, many countries have ongoing efforts to strictly protect additional forest regions, such that our assessment would require future updates. However, as of 2024, we believe this to be the most up to date assessment of strictly protected forests in Europe that includes data on sizes of protected forest areas.

Although we tried to adhere to the general criteria for inclusion in the database (i.e. forest cover; no timber extraction; ≥ 5 ha; and some form of legal protection), we did allow for exceptions in some cases. Such cases included removal of dangerous trees along hiking trails, fire suppression measures when necessary, and some rare cases of sanitation cuts (described in detail for each country below). Moreover, due to various data constraints, the data for each country were calculated using a variety of approaches that are not consistent across every country. In most cases we provide sources for retrieving original data, but in some cases the data is not publicly available and was sourced through direct communication with national agencies. Data on the total forest cover for each country was taken from the 2020 country reports from the FAO Global Forest Resources Assessment. Detailed descriptions of the methods used for each country are provided below.

Finally, as pointed out in the manuscript, data on the amount of extensive and intensive forest management in each country was extracted from Mason et al. (2021). However, for countries not included in their study, or when updated data were available, we describe this for relevant countries below.

Frank, G., Parviainen, J., Vandekerhove, K., Latham, J., Schuck, A., Little, D., (Editors), 2007: COST Action E27 Protected Forest Areas in Europe – Analysis and Harmonisation (PROFOR): Results, Conclusions and Recommendations. Federal Research and Training Centre for Forests, Natural Hazards and Land- scape (BFW). Vienna, Austria. 211 p.

Mason, W.L., Diaci, J., Carvalho, J. & Valkonen, S. (2021). Continuous cover forestry in Europe: usage and the knowledge gaps and challenges to wider adoption. *Forestry: An International Journal of Forest Research*, 95, 1–12.

Parviainen, J., Bücking, W., Vandekerkhove, K., Schuck, A. & Päivinen, R. (2000). Strict forest reserves in Europe: efforts to enhance biodiversity and research on forests left for free development in Europe (EU-COST-Action E4). *Forestry*, 73, 107–118.

Austria

Forest ecosystems are protected within several different types of protected areas in Austria, including the natural forest reserve network, steppingstone habitats, and core areas of national parks. The following description provides details on the data sources and methods used to quantify strictly protected forests areas in Austria. Date on the proportion of even-aged and uneven-aged forests in Austria was based on the national NFI data.

Input Data

Table1: Input Layers:

Area Type	Dataset	Source/Link
Protected Areas	**Natural Forest Reserves (NFR)**	http://www.naturwaldreservate.at/index.php/de/
Protected Areas	**Steppingstone Habitats (StSt)**	https://trittsteinbiotope.at/
Protected Areas	**Protected Areas in WDPA** (World Database of Protected Areas (2021)	https://www.protectedplanet.net/country/AUT
Forest Area	**Forest Layer, spatial resolution 1x1 m** - BFW (2018)	https://www.bfw.gv.at/hochgenaue-waldkarte-waldinventur/

Natural Forest Reserves:

Natural forest reserves (NFR) are forest areas that are intended for the natural development of the forest ecosystem and in which there is no timber extraction, other forestry utilisation, or anthropogenic influence. They contribute to the preservation of the natural development of biological diversity. Hunting is permitted - even necessary, as the reserves can otherwise represent resting areas for hoofed game.

They are used for research, teaching and education (Federal Ministry of Agriculture and Forestry 1995: Forestry principles of the federal government for the establishment of an Austria-wide network of natural forest reserves). The selection of NWRs is primarily orientated towards the occurrence of potential natural forest communities (PNWG). They are intended to represent the tree species composition, stand structure, vegetation and, in particular, the natural development of these or to reach these in the foreseeable future.

In Austria, there are a total of 118 forest communities relevant to the NWR programme in 22 growth areas. Each forest community occurring in these 22 growth areas is to be covered by at least one reserve. The aim is not to preserve the current state, but to allow natural development.

There are currently 191 natural forest reserves with a forest area of 8,587 ha (as of March 2021).

Steppingstone Habitats

These include small areas within a forest that are taken out of management. They are established in the context of two projects at BFW: ConnectForBio and ConnectPlus.

Stepping stone biotopes with an area of 1.5 ha or more are created and accompanying scientific studies on microhabitats and various species groups are carried out. In order to maximise the networking effect, priority areas and suitable sites are designated, taking into account the forest development plan.

A contractual agreement between forest owners and the Austrian Research Centre for Forests (BFW) regulates the paid external use for a certain period of time, during which the forest owners may neither reforest nor remove trees. No other interventions are permitted either. However, hunting management is an exception.

Processed Data

Filter on Protected Areas:
All three spatial datasets from the protected area classes (WDPA, NFR, StSt) were filtered according to the following criteria:

- Area > 5 ha
- National forest reserve status = 1 (established by contract)
- WDPA Category = Ia OR Ib OR II
- Steppingstone status → established by contract
  → this resulted in 10 Steppingstones, 173 natural forest reserve areas, 34 WDPA regions.

Processing:
The protection areas were combined with the forest area to determine the forest cover. Protected areas without forest cover were taken out of the list. For the others, forest cover was calculated with a precise and high-resolution forest cover map created by BFW in 2018. The steps were the following:

- Merging all three filtered and protected layers
- Calculated zonal statistics with each protected area calculating the total forest cover per area (pixel-Sum = m^2)
- Divide by 10,000 → hectares
- Create percentage of forest cover for each protected area

ID creation
In order to protect private information, we created new IDs for the stepping stone habitats and the NFR:

- Create new ID to identify each singular protected area from the 3 datasets (data protection)
- <u>WDPA:</u> "NAME" || ', WDPAID: ' || to_string("WDPAID")
- <u>NFR:</u> 'Natural Forest Reserve ' || to_string("fid")
- <u>Stepping Stones:</u> 'stepping stone biotope ' || to_string("fid")

Bosnia and Herzegovina

The network of protected areas in Bosnia and Herzegovina is still under development. As of 2020, there were 153 areas under some form of protection in BiH, with different definitions and forms of governance in the Federation of Bosnia and Herzegovina or the Republika Srpska. However, only a small fraction of these protected areas include strictly protected forest ecosystems without any management (Report, 2016). These include the old-growth forests Janj, Lom, and Perućica, within the Republika Srpska. In our assessment here, we also included a small number of unmanaged old-growth remnants that are protected within local forest management units (Monograph, 2019).

Strategy and Action Plant for Protection of Biological Diversity (2015-2020). 2016. Ministry of Foreign Trade and Economic Relations.

Virgin forests of Bosnia and Herzegovina. 2019. Sarajevo, ISBN=978-9926-8245-4-9, publisher: Forestry Association of Federation of Bosnia and Herzegovina. 249 pages.

Bulgaria

The Bulgarian system of nature protection is based on several types of protected territories. Some of them are strict reserves that exclude management, while others allow management under some restrictions and specific rules. There are 55 strict forest reserves (IUCN category Ia, total area approx. 77000 ha). These territories are owned by the government. Any management is forbidden. In extreme cases such as large and expanding wildfires, threatening a large territory of the reserve, there could be an exception for limited activities for fire suppression. This has happened several times in the last two decades. After fires it is not allowed to do any salvage logging or extraction of dead wood. For composing the present dataset, only territories of strict forest reserves were used. The forested area of the reserves was calculated as the sum of the areas of the forest stands as indicated in the forest

inventory. Where there was no forest inventory, polygons were drawn on the non-forested areas and their area were subtracted from the total area of the reserves.

Other types of protection categories are present in Bulgaria that have very little human influence. Although they were not included in this assessment, we briefly describe them below.

The other type of protected territories with almost complete exclusion of management are National Parks. They are also completely government-owned and correspond to category II of IUCN. Forest management activities could be allowed only to a very limited extent, such as collection of small amounts of dead wood close to huts or roads. However, in recent decades this was very limited. No salvage logging or other management systems are allowed. Human activities are limited to tourism, following strict regulations and allowed mostly in specifically designed areas for tourism. The type of tourism is mostly hiking except for a few areas with ski facilities. Most of the Nature park territories are in zones for nature protection and for limited human presence, which are similar to the management approach in strict reserves. There are 3 national parks with total territory of 150360 ha (1.35% of the territory of Bulgaria). More than half of their territory is with alpine character. The protected forests are mostly coniferous (Norway spruce, Silver fir, Scots pine, Macedonian pine, Dwarf mountain pine, Bosnian pine and Beech (either pure or mixed with conifers, situated mainly in Central Balkan National Park). Strict forest reserves are included in the territory of the National Parks. More intensive interventions in these forests in the last three decades were related mostly to suppression of large fires. No salvage logging was allowed after such activities.

The other type of protected area is Nature parks. There are 11 with a total territory of 264551 ha. Most of it is forested. Nature parks allow for forest management, but no salvage logging is permitted. Forest management is extensive and limited. It is regulated by the Nature park management. A specific type of protected area is the Maintained reserve. There are 35 with a total area of 4521 ha. These are mostly small areas with a certain type of ecosystem, which might require specific management activities to maintain its character. Forest management is extremely limited and extensive, allowed only in the cases when it will support a certain threatened species or ecosystem, which is the main protection target.

Finally, a large fraction of the forested areas of Bulgaria (more than 60%) are included in NATURA2000 areas. However, most of them still do not have management plans (as to September 2023). As a general rule, management should not damage the natural forest composition expected for the specific ecosystem. No large-scale logging is allowed. Plantations, where existent, should be managed so foreign species should gradually be excluded and replaced by local species, mostly following natural transition by regeneration. New plantations are limited and they should be with local species, expected for the specific ecosystem.

To determine the amount of extensive and intensive managed forest, we used data from the Bulgarian State Forest Agency for distribution of forests by dominant species, and interpretation based on expertise.

Croatia

The main protected areas of Croatia are National parks, Nature parks, and Strict reserves. There are 444 protected areas in Croatia, encompassing 10% of the country (5,930 km^2). Other protected nature categories are special reserves, regional parks, natural monuments, important landscapes, forest parks and monumental park architecture. All nature protection activities are managed by the State Agency for Nature Protection.

Certain protected areas have been included in the international system for nature protection. The Plitvice Lakes and, since 2017, two primeval beech forests on Mount Velebit are on the World Natural Heritage List; Mount Velebit and the Mura–Drava regional park (part of the cross–border Mura–Drava–Danube biosphere reserve) belong to an international network of biosphere reserves (Man and the Biosphere – MAB), while Kopački Rit, Lonjsko Polje, the Neretva Delta, Crna Mlaka and Vransko Lake are on the international list of valuable wetlands (Ramsar Convention). The Papuk Nature Park is part of the European network of geoparks.

Strict forest reserves, including old-growth forests, are mostly protected in the categories of special reserves of forest vegetation. Most of them are located within larger protected areas such as National Parks and Strict reserves. For these currently existing forest reserves, management is

prohibited. It is worth noting that great potential (e.g. mature forests or secondary old-growth) for creating new strict protected areas exists in commercial forests as well as in subalpine vegetation zones where there was no intensive management in the past. Currently, changes are being made to the law on nature protection, where categories and definitions of primary and old-growth forests are introduced, and a working group was established to select new high-value forests with the character of reserves.

Further information on the protected areas of the Republic of Croatia can be found on the Bioportal website (www.bioportal.hr/gis) and via WMS and WFS web service. These sites contain information on protected areas in the Republic of Croatia in the protection categories according to the Nature Protection Act: strict reserve, national park, nature park, special reserve, regional park, nature monument, significant landscape, park — forest and monument of park architecture.

Czech Republic

The Czech Republic has several types of protected areas with different degrees of protection. Strict protection excluding forest management is obligatory only in core parts of national parks and some part of nature reservations. Therefore, the list was compiled from strictly protected areas delimited by the offices of the respective national park and complemented by the set of the nature reservations in which strict forest protection is declared. In some other nature reservations, strict forest protection is required by the law since 2017, however, the management measures are allowed on condition that they targeted protection of particular species.

Denmark

The dataset for Nature Forest Reserves in Denmark is derived from two sources depending on forest ownership. The areas considered are forest reserves where timber production has been permanently abandoned. However, certain management and restoration activities with the purpose of enhancing biodiversity are allowed in the initial years following abandonment.

The data from the state forests was obtained from the Nature Agency of the Ministry of Environment. Due to the ongoing designation of nature reserves in Denmark, the official map has not been made public yet.

Data from private forests consists of a map of areas included in a subsidy program from the Danish Nature Agency. Under this program, private forest owners receive payment for permanently setting forest aside under the same conditions as the public forests. The maps contain the areas included in the program up to 2020 and can be downloaded from: https://miljoegis3.mim.dk/spatialmap?profile=privatskovtilskud

The forest maps are derived from stand-level management mapping of forests, and can result in many small polygons due to different management regimes (i.e. different tree species/age), even when they correspond to the same forest complex or adjacent to each other (e.g. separated by a forest road). Therefore, forest polygons within a distance of 5 m or less to each other were aggregated.

Estonia

The Estonian protection system includes various types of area protection, which are not necessarily spatially exclusive. For example, there may be a strictly protected habitat or a key habitat (requiring strict protection) within a multipurpose protection zone; in that case the stronger requirements apply to a particular place within the zone. Given that, the current list has been compiled based on maximum national protection regime of each patch.

This list follows strict protection in terms of forestry (no timber harvesting allowed). Such patches are not necessary strict reserves in the IUCN terms; e.g. hunting may be allowed. Also, many reserves comprise patches that are spatially apart (even kilometres), which ecologically serve as distinct reserves. The list was compiled using a separate GIS procedure based on two officially available spatial databases:

- the protection regimes as shown in the Estonian Nature Information System (as of 26.10.2022)

- the forest areas as shown on the Estonian Base Map of the Estonian Land Board (as of 7 April 2021).

A detailed description of forest definition of the base map in Estonian can be found here: https://geoportaal.maaamet.ee/index.php?lang_id=1&action=kataloog&tyyp_id=15&page_id=88 In general it requires at least 0.5 ha size and at least 30% canopy cover, but it also includes forest rides within forest tracts and clearcuts.
These databases were intersected in the following procedure:

- only a single strictly protected layer was created (based on maximal protection and removing overlapping regimes);
- adjacent patches up to 50 m from each other have been treated as a distinct *reserve*, independently of whether they were registered under a common protected area name or not;
- each of these *reserves* was given a unique ID and its total area was updated;
- all *reserves* <5 ha in total size were removed;
- for the remaining *reserves*, the total intersecting forest area was calculated;
- only the *reserves* that have >1 ha forest were retained.

In total, 2865 *reserves* were retained on the list, with a total area 596,424 ha and total forest area 302,924 ha.

Finland

The Finnish data for reserves contains only forests that are strictly protected from harvesting, based on the situation in the beginning of 2022. Restoration measures may be possible, but not forestry operations. In the national statistics, this includes categories 1A, and the part of category 1B that is permanently outside of forest management. Category 1A consists of nature reserves and sites reserved for nature conservation. Category 1B consist of other statutory protected areas.
It should be noted that the values for the total area of forestry land (productive and poorly productive land, growing > 0.1 m3/ha/year) is a sample-based estimate from the National Forest Inventory data. Here, the data comes from the 12th and 13th inventory cycles (years 2016-2020).
The data was extracted from the Statistic Database of Natural Resources Institute of Finland. Data on the total amount of forest land is available at:
https://statdb.luke.fi/PxWeb/pxweb/en/LUKE/LUKE__04%20Metsa__02%20Rakenne%20ja%20tuotanto__04%20Metsien%20suojelu/01_metsien-suojelu.px/

Data on the conservation areas is available at:
https://statdb.luke.fi/PxWeb/pxweb/en/LUKE/LUKE__04%20Metsa__02%20Rakenne%20ja%20tuotanto__04%20Metsien%20suojelu/01_metsien-suojelu.px/

The data is described in this document (in Finnish): https://www.luke.fi/fi/tilastot/metsien-suojelu/metsien-suojelutilaston-laatuseloste
All links were last accessed Sep 18, 2023.

Size distributions of forest reserves

Similar, readily available statistics on the sizes of individual forest reserves and the area of forests were not available. To obtain this information, we used the nature protected areas and wilderness reserves polygon dataset, compiled and provided by the Finnish Environment Institute, and Corine 2018 land cover data.

Protected areas include many different types of protected areas, and larger areas divided by roads, rivers or peatlands that are in the database separated into adjacent polygons. However, from the point-of-view of forest-dwelling organisms these form contiguous areas and were hence combined here.

As a starting point, we took the three protected area datasets: state-owned protected areas, private protected areas, wilderness areas, and merged all three into one. We then merged all neighboring polygons (i.e., polygons that shared at least some border). To account for protected areas that in the

original polygons were dissected by roads, rivers or other similar features, we then further combined areas that were less than 50 m apart. This then resulted into contiguous protected area polygons, regardless of ownership or conservation program. To obtain the size distribution of protected areas, we then calculated the sizes of these areas, and removed those smaller than 5 ha.
Then, we calculated the total number of Corine cells in each polygon, and the total number of forested Corine cells, which we then used as the proportion of forests in each polygon. We then obtained the forest area by multiplying the total area of polygons with the proportion of forests. Finally, we removed polygons with less than 5% forests, to remove e.g., large marine reserves that might hold some small islands, etc.
Data sources (accessed Sep 18, 2023):
Nature protected areas and wilderness reserves dataset:
https://ckan.ymparisto.fi/dataset/%7BC8FC4A42-A2C3-40C4-92CD-2299C688514E%7D
Corine land cover 2018: https://ckan.ymparisto.fi/dataset/corine-maanpeite-2018

France

High level of protection (IUCN Ia) in France comprises two types of areas:

- Nature reserves, the management of which is decided during the management plan construction. It may comprise strict protection (harvesting abandonment) as management types. The data included in the database are issued from the report of Cateau et al (2017) that inventoried all nature reserves for which strict protection was included in the management plan. This inventory dates back to 2013 and has not been updated since;
- Strict forest reserves are managed by the national forest service (public forests) and designated upon ministerial decision on the proposal of local services. The areas designated are then transmitted to the National Landscape and Nature Inventory, from where the data has been extracted (https://inpn.mnhn.fr/programme/espaces-proteges/protections-reglementaires).

Cateau, E., Duchamp, L., Garrigue, J., Gleizes, L., Tournier, H., Debaive, N. (2017). Le patrimoine forestier des réserves naturelles. Focus sur les forêts à caractère naturel. Cahier des Réserves Naturelles de France 7, nov. 2017. 104p.

Germany

The determination of a reliable value for the extent of non-intervention forests in Germany that meet the criteria of strict forest reserves is complex. In Germany, forest reserves can be secured through various instruments and can be found in all forest ownership types. Among others, strict forest reserves may lay in Natural Forest Reserves (Naturwaldreservate), in the core zones of National Parks and in the core zones of Biosphere Reserves. As it was not possible to obtain complete data for these three protected area categories, this study uses an aggregated value for forests with natural forest development provided by the NW-FVA.

Forests with natural forest development (NWE) can be understood as an umbrella category of non-intervention forests. In the sense of the National Biodiversity Strategy, NWE stands for forests or areas suitable for forest cover of at least 0.3 ha, on which neither forestry nor nature conservation or landscape management interventions take place. The renunciation of use needs to be permanent and legally binding (Table 1). Due to continuing legal obligations, interventions may become necessary on NWE sites in certain situations. These include, in particular, measures to ensure public safety, fire and forest protection measures in case of imminent danger and hunting/wildlife management. Public access and research activities are mostly permitted.

Table 1. Criteria for forests with natural development. The definition is based on standards by MCPFE- and IUCN as well as a discussion process between experts during the research project „NWE5“.

**Minimum requirements for forests with natural development**

Definition	Forest or areas suitable for forest cover on which neither forestry nor nature conservation or landscape management interventions take place and for which this has been legally established on a permanent basis.
Minimum area	≥ 0.3 ha
Legally binding assurance of permanent non-use	Sovereign public law, contractual or in rem safeguarding of permanent natural forest development.<br>Examples: Decree/ ordinance as a protected area, contract-based nature conservation, compensation measure, land register entry, published nature conservation concept as documented self-commitment, foundation statutes.
Current forest stand	Closeness to nature of the current stand is desirable from a nature conservation perspective, but not obligatory in terms of the minimum requirements. Forest succession areas (e.g. abandoned military training areas) are also eligible for recognition.
**Permitted measures on NWE sites**	
Forest protection	Hunting, fire protection and forest protection measures in case of imminent danger
Recreation	Public access, measures to ensure public safety (Verkehrssicherung)
Research	Non-destructive research activities

The following information on the area of forests with natural development are derived from the so-called "NWE5" database, which is administered at the Northwest German Forest Research Institute (NW-FVA). The NW-FVA last accounted the nationwide NWE area in 2020 by means of a standardised data query with relevant stakeholders. As of December 2020, Germany's NWE area was 355,122 ha (Steinacker et al. 2023). Please note that the stated numbers for forests with natural development contain non-intervention forests located in natural forest reserves, national parks and biosphere reserves, among others. The aggregated NWE value must therefore not be added to the hectare values from other protected area categories.

As the minimum requirements for NWE areas differ from the proposed definition of strict forest reserves used in this study, this total was filtered. About 5 % of the NWE areas (18,701 ha) is smaller than 5 ha (Table 2). Furthermore, for about 15 % of the declared area (55,212 ha), no information is available on the individual area size. Assuming that the latter are likely to be ≥ 5 ha, a total of **335,618 ha** meet the criterion of minimum area size for strict forest reserves.

Table 2: Size distribution of the individual NWE areas in 2020. Information was available for about 85 % of the area with natural forest development. Neighbouring polygons separated by less than 40 m were counted as one continuous area.

**Size category of NWE**	**area [ha]**	**proportion of size class within NWE network [%]**
0.3 – 5 ha	18,701 ha	6.3
5 – 20 ha	26,283 ha	8.8
20 – 100 ha	58,388 ha	19.5
100 – 500 ha	56,581 ha	18.9
500 – 1000 ha	23,638 ha	7.9
> 1000 ha	115,495 ha	38.6

According to the criteria for this study, strict forest reserves require an official protection status by law. The NW-FVA, on the other hand, also considered other types of safeguarding as legally binding for their analysis on natural forest development (Table 1). In addition to sovereign instruments, NWE can

thus be secured by land register entries, long-term contractual agreements (e.g. National Natural Heritage, contractual nature conservation, permanent compensation measures) and self-binding commitments. The self-binding commitments need to be documented, for example in the form of published nature conservation concepts, the statutes of a foundation or a resolution by a local council.

Finally, the sizes of individual reserves are not publicly available due to privacy considerations, and are not included in the open access database that accompanies this study. However, they are included in all the analyses.

Steinacker, C., Engel, F., Meyer, P. 2023. Natürliche Waldentwicklung in Deutschland: auf dem Weg zum 5 %-Ziel der Nationalen Strategie zur biologischen Vielfalt. Natur und Landschaft 98: 545-552.

Greece

Strict nature protection zones

Areas designated as strict nature protection zones are characterized by exceptionally sensitive ecosystems, habitats of rare or threatened species of indigenous flora or fauna, or areas that hold significant positions in the life cycle of rare or endangered wildlife species. These areas are strictly monitored and protected by the competent authorities. All activities are prohibited within strict nature reserve areas. However, exceptions may be allowed, according to specific regulations outlined in the respective management plan, for conducting scientific research while ensuring a high level of protection. Additionally, essential activities that are deemed necessary to maintain the characteristics ensuring the preservation of protected objects, species, or habitats may be permitted*

* Selective tree removal for Wildlife Habitat Improvement (in practice)

Nature protection zones

Nature protection zones are defined as areas with types of natural habitats and/or species features whose presence and representativeness are assessed as high, or whose condition requires strict protection. In these zones, the natural environment is protected from activities or interventions that could significantly worsen its physical condition, composition, or development. According to the specific regulations of the designation act of the protected area and/or the relevant Management Plan, activities that undermine the management objectives or the effectiveness of the management measures of the protected area are prohibited or restricted. Only certain or all the special categories of uses provided for in Article 14b of PD 59/2018 are allowed in the nature protection zones.

These special uses are selected and may be specified case by case for each protected area, based on the specific environmental study of paragraph 2 of Article 21, according to the presidential decree of paragraph 4 of Article 21*

* Agricultural operations, facilities, and activities
* Public Transportation Facilities

Habitat and Species Conservation Zones

Habitat and Species Conservation Zones are defined as areas subject to appropriate management to ensure a satisfactory level of conservation for the protected objects (types of natural habitats and species of Union significance and/or national interest) they host. Activities within Habitat and Species Management Zones are prohibited or restricted according to the specific regulations of the designation act of the protected area and/or the relevant Management Plan when they, individually or cumulatively with others, can degrade the conservation status of the protected object. This is especially true when such degradation negatively impacts the conservation status of the protected object at the national level.

Only certain or all the special categories of uses provided for in Article 14γ of PD 59/2018 are allowed in habitat and species conservation zones. These specific uses are selected and may be specified case by case for each protected area based on the specific environmental study of paragraph 2 of Article 21, according to the presidential decree of paragraph 4 of Article 21*

*(Extractive activities (Mines, Quarries, Mines, Sand extraction, Exploration and Exploitation zones of hydrocarbons).

Zones of Sustainable Natural Resources Management

These zones are defined as protected areas where the protected object can coexist with relevant cultural values or human activities that promote the sustainable management of natural resources or sustainable development. This type of management serves environmental protection, economic development, social cohesion, and addressing climate change issues. Human activities within this zone that could lead to the degradation of the degree of conservation of the protected object in the protected area, especially at the national level, are subject to appropriate regulations based on the relevant provisions of the designation act of the protected area and the respective Management Plan.
Certain or all special categories of land use as per article 14d of PD 59/2018 (Government Gazette A' 114) are allowed in zones of sustainable natural resources management. These specific uses are selected and may be specified on a case-by-case basis for each protected area, based on the special environmental study of paragraph 2 of article 21, through the presidential decree of paragraph 4 of article 21*
*Timber harvesting

In our comprehensive examination of the strict forest reserves in Greece, our research specifically focused on the delineated 'Strict Nature Protection Zones.' These zones hold paramount importance due to their designation as areas of exceptional sensitivity encompassing critical ecosystems and habitats that are home to rare or endangered indigenous flora and fauna. Within these areas, any form of human activity is strictly prohibited by law, marking them as sites of non-interventionist preservation. However, it's essential to note that specific regulations outlined in the respective management plans may allow for exceptions, particularly for conducting scientific research, provided it aligns with stringent guidelines ensuring the utmost protection of these delicate ecosystems.

Contrasting these strict nature reserves are other protected area categories within the Greek legislation framework, each with varying degrees of permissible management interventions. For instance, Nature Protection Zones encompass areas with high-value natural habitats or species requiring strict protection. While interventions in these zones are restricted to prevent significant deterioration, certain specific activities, such as agricultural operations or public transportation facilities, may be permitted under defined regulations outlined in the designation act and the relevant Management Plan.

Similarly, Habitat and Species Conservation Zones aim to conserve specific habitats and species of Union significance and/or national interest. In these zones, activities that might degrade the conservation status of the protected objects are prohibited or restricted, with selective uses, such as extractive activities, being considered only under specific environmental assessments and regulations set by the Management Plan.

Additionally, Zones of Sustainable Natural Resources Management represent areas where protected objects coexist with cultural values or human activities promoting sustainable natural resource management or development. While these zones allow certain activities like timber harvesting, they operate under stringent regulations to prevent any degradation of the protected object's conservation status.

Data provided in this study focused exclusively on the Strict Nature Protection Zones, acknowledging their significance in preserving untouched and ecologically vital forest ecosystems within Greece. By concentrating on these strictly preserved areas, we aimed to provide a precise assessment of forests devoid of any form of human exploitation, contributing to a more nuanced understanding of the preservation efforts and ecological richness within these unique zones.

Hungary

In Hungary the entire forested area is officially 1 938 544 ha (based on forestry plans, National Forestry Database, 2016 – Kolozs and Szepesi 2010). There is a range of legislation (both national and European) providing some level of protection for forests but most of these cannot be considered strict as they allow different levels of management activities. According to the criteria used in this study, there are two types of protection which we consider as a strict forest reserve. The first is the core area of

forest reserves, of which there are 63 in the country, covering 3665 ha (https://erdorezervatum.hu/). Forest reserves in Hungary are not simply non-managed forests but also designated sites for research and the long-term monitoring of natural forest development processes. All reserves consist of a buffer zone and core area – but continuous cover forestry management is allowed in the buffer zone. The second type are the unmanaged or set-aside forest, defined by the forestry system “without timber production purposes”. In these forests there is no timber production for different reasons, such as sensitivity to erosion, low productivity, conservation purposes, or difficult accessibility (protection forests). The area of these forests is 82 235 ha, but all management activities (including sanitary cutting) is prohibited on only 29 474 ha, which were included as forest reserves for this study. Altogether the area of forest reserves (strictly protected forests) is 33 139 ha (1.71% of the total forest area). The number of these sites is 2521, their mean size is 13 ha. 26 233 ha (1.35%) of the Hungarian forests are managed in continuous cover forestry system while 73 449 ha (3.79%) in transitional forestry system. These two management types (forestry systems) are considered as extensive in this study (99 682 ha, 5.1%). All other forests are managed by rotation forestry system, regenerated artificially (clear-cutting) or naturally (shelterwood system).

Kolozs, L., Szepesi, A. 2010. Chapter 16 – Hungary. In: E. Tomppo, T. Gschwantner, M. Lawrence, R.E. McRoberts (Eds.), National Forest Inventories: Pathways for Common Reporting, Springer, Dordrecht, pp. 269-276, https://doi.org/10.1007/978-90-481-3233-1.

Italy

Currently, 21.4% of terrestrial area of Italy is designated as protected areas (source: https://biodiversity.europa.eu/countries/italy [Accessed: 06 February 2024]). Italy has a total of 3,512 protected areas, comprising 875 sites designated under national laws and 2,637 recognized as Natura 2000 sites. The majority (37%) of terrestrial protected areas in Italy are 1-10 km^2 in size and the proportion of smaller protected areas (less than 1 km^2) is 24%, which is lower than the EU average. About 2% of terrestrial protected areas are larger than 1,000 km^2.

A complex network of national and regional protected areas (PAs) designated under the National Law 1991/394 overlaps with over 50% of the Natura 2000 sites (Marchetti et al., 2005). National PAs include national parks, state nature reserves, and other national protected areas; regional Pas include regional parks, regional nature reserves, and other regional and local protected areas. Each category of PA has common general aims and protection status, and they are managed by environmental plans, the aims of which frequently coincide with the requirements of the Birds and Habitats Directives (Trentanovi et al., 2018). Moreover, according to National Law 1991/394, every Italian Region has its own legislation for regional and local PA recognition and management.

Regardless of their national or regional level or protection status, each environmental plan should have its own zoning schemes which define different protection level of zones within the PA boundary, from the so-called ‘core areas’ (even if they are identified with heterogeneous definitions based on the different regional legislation), where strict nature conservation is enforced, to zones where gradually more intensive human presence and activities are allowed.

Methods

First phase: data recognition

Data was collected by downloading the available geospatial vector data (i.e., protected areas boundary, protection status and eventual zoning) from regional and national geoportals; subsequently, lacking and not available data were requested from regional and national authority.

After this preliminary data recognition, we were able to reach the following scenario (updated to January 2023):

1. The boundaries of PAs are available in geospatial vector format for most of the cases. Some geoportals report the updated list and boundaries of the protected areas (compared to the National List of Protected Natural Areas - EUAP - which has not been updated since 2011);

2. PAs zoning (i.e. division within different level of protection within each PA, from the strictly protected 'core areas' to areas where human activities are allowed with different intensity levels) is fully available in digital format only for the PAs of 3 regions (out of 21).
3. Zoning is entirely lacking for some protected areas (e.g. zones still need to be identified by the planning authorities).

Second phase: data elaboration
Based on the above-mentioned evidence and gaps, we decided to adopt the following approach to reach a fair approximation of Italian strict protect forests:

- where zoning data was available, we considered strict protected forests (i.e. those overlapping in the 'core areas') those falling in the zone 'A' or '1' for Regional or National reserves and in zone 'A' or RN) for Regional or National parks;
- in the cases where zoning data was not available, we applied some reasonable thresholds to determine a strict protected area percentage for the different types of PA: for regional or national reserves, we considered 50% (except for those reported in the attributes as 'riserva integrale' or 'riserva biogenetica' or 'area di rilevante interesse naturalistico' considered as 100%) of the forest area falling into the whole protected area. In case of overlapping sites (reserves included in larger parks) it was counted only the strict reserve area. For regional (and provincial) or national parks it was considered 30% of the forests falling into the whole PA. These percentages are based on the observed proportion of core areas defined in the significant sample of PAs where zoning data were available;
- for the other small naturalistic sites (e.g. natural monument, natural interest, biotopes, oasis) we considered 100% of the forests falling into the whole protected area. We did not consider the 'area contigua' of regional and national parks as well as recently designed landscape protection areas (e.g. 'Zona di Salvaguardia' in Piemonte or 'Paesaggio protetto' in Emilia-Romagna, 'Area naturale di Interesse Locale' in Tuscany, 'Parchi comunali e intercomunali' del FVG, 'Parco territoriale attrezzato' in Abruzzo) or elements of the regional ecological network (e.g. corridors) outside protected areas.

Marchetti, M., Cullotta, S., Di Marzio, S. (2005) I sistemi di aree protette in Italia e il loro contributo alla conservazione forestale. L'Italia Forestale e Montana,60(4), 559–581.

Trentanovi G., Campagnaro T., Rizzi A., Sitzia T. (2018) Synergies of planning for forests and planning for Natura 2000: evidences and prospects from northern Italy [Special issue: Forest management and Natura 2000 in the alpine and continental biogeographical regions: bridging research and practice]. Journal for Nature Conservation, 43: 239–249. DOI 10.1016/j.jnc.2017.07.006

Latvia

Specially protected nature territories in Latvia are geographical areas that are under special state-level protection, in order to safeguard and maintain biodiversity of nature – rare and typical ecosystems, habitats for rare species, landscapes, that are peculiar, beautiful and characteristic for Latvia, geological and geomorphological formations, as well as territories, significant for recreational and educational purposes.

National parks are broad areas which are characterised by outstanding nature formations of national significance, landscapes and cultural heritage landscapes untouched by human activities or nearly natural, a diversity of biotopes, abundance of cultural and historical monuments, and peculiarities of cultural environment.

Nature parks are territories that represent the natural, cultural and historical values of a particular area, and that are suitable for recreation, education and the instruction of society. Organisation of recreation and economic activities in nature parks shall be carried out by ensuring the preservation of the natural, cultural and historical values located in such parks.
Protected landscape areas are territories remarkable for original and diverse landscapes and special beauty. The goals of such territories are to protect and preserve the cultural environment and landscapes characteristic of Latvia in all their diversity, as well as to ensure the preservation of environment appropriate for recreation of society and for tourism, and use of environment friendly management methods.
Nature reserves are nature territories little transformed or transformed in varying degrees by human activities, which territories include habitats of specially protected wild plant and animal species, and specially protected biotopes.
Strict nature reserves are territories untouched by human activities or nearly natural, in which territories unhindered development of natural processes shall be ensured in order to protect and study rare or typical ecosystems and parts thereof. Strict nature reserves shall have zones in which all natural resources are completely excluded from economic and other activities.
Microreserves are territories that are designated for the protection of especially rare species and their habitats. In microreserves similarly to specially protected natural territories certain actions that may threaten the rare species or their habitats are restricted or prohibited. Microreserves are usually smaller than specially protected areas (0,1 – 20,0 ha, for birds up to 500 ha) and the establishment procedure is less complicated and shorter as than that of specially protected areas. As microreserves are established for the protection of especially rare species, the information regarding the microreserves and the species found in them is stored in the Data Base of Nature Conservation Agency and is available for registered users only.

Each of the specially protected areas have zoning with different restrictions level. Only zones where all forest management activities are forbidden and which meet the specified criteria in this study were included in the list. The general information about specially protected areas was obtained via the Nature conservation Agency Republic of Latvia; https://www.daba.gov.lv/en/protected-areas, yet more specific information about territories where forest management activities are forbidden and which meet the criteria was obtained through direct communication with the State Forest Service.

Lithuania

The protection area system in Lithuania is vastly different from those of the west (Angelstam et al. 2021), has a long, complicated history and is based on previous Soviet forest zoning strategies (Brukas, 2015). From the middle of the 1990s, Lithuanian Forest Management has applied a triad approach by creating four forest management function groups; Group I strict nature reserves, Group II which is divided into (A) ecosystem protection, and (B) recreational, Group III protective, and Group IV commercial forests (Parliament of the Republic of Lithuania, 2010). Each group has a predetermined set of economic, social and conservation and protection goals. This means that if a protected forest area falls within a National or Regional Park, then the forest land is primarily managed under the regulations of the Lithuanian Forest Services. Thus, there is often conflicts between the State Forest Service and the Protected Area Service authorities.

Accordingly, Group I (strict nature reserves) are forests that are segregated for the strict protection of nature. The area of strict protection forest reserves covers 1.2% of the forest area. Within this group forest harvesting is prohibited.

Group II forests can be considered as multifunctional (extensive) forests that combines nature protection and forest harvesting by utilising a set of higher forest harvesting regulations (i.e., increased harvest ages and the use of continuous cover forest management). This forest group cover 11.9% of Lithuania's forest area.

Group III are protective forests managed that aim to protect soil, air, water functions and cover 14.6% of Lithuania's forest area. Forest harvesting regulation include an increased harvest age and clearcut size can be no larger than 5 ha (Parliament of the Republic of Lithuania, 2010). It should be

noted that there is confusion within the forest sector as to what forest management activities can be undertaken in the Group II and III forest, so more often than not they are treated as commercial forests.

Group IV forest are for commercial purposes and cover 72.3% of Lithuania's forest area. These forests are subject to clearcut forest harvesting once they reach the nominated harvesting age (see Table 1) and clearcut sizes can be no larger than 8 ha (Parliament of the Republic of Lithuania, 2010). Both group IV and III forests are considered as intensively managed forests (State Forest Service 2022).

Table 1.Minimum harvest ages of stands for each forest functional group (Parliament of the Republic of Lithuania, 2010).

**Tree species**	**Group IV forests**	**Group III forests**	**Group II forests**
Scots Pine, Larch, Ash, Maple, Beech, Elm	101	111	171
Norway Spruce	71	81	121
Oak	121	141	201
Birch, Black alder	61	61	91
Small leaved lime, Hornbeam	61	71	91
Aspen	41	41	81
Willow sp.	31	31	51

To identify the strict protection forest reserves in Lithuania, we used the Lithuanian Forest Stand Cadastral Geospatial Database (Lithuanian State Forest Service 2021) by selecting forest data based on the four forest management function groups. In addition, we also intersected the official geospatial database of the State protected area cadastre (https://saugoma.lt/en/) to help identify the protected area names.

Angelstam, P.; Manton, M.; Yamelynets, T.; Fedoriak, M.; Albulescu, A.-C.; Bravo, F.; Cruz, F.; Jaroszewicz, B.; Kavtarishvili, M.; Muñoz-Rojas, J.; et al. Maintaining Natural and Traditional Cultural Green Infrastructures across Europe: Learning from Historic and Current Landscape Transformations. Landsc. Ecol. 2021, 36, 637–663.

Brukas, V. New World, Old Ideas—A Narrative of the Lithuanian Forestry Transition. Journal of Environmental Policy & Planning 2015, 17, 495-515, doi:https://doi.org/10.1080/1523908X.2014.993023.

Parliament of the Republic of Lithuania. On the Approval of Regulations for Forest Fellings: The Order of the Minister of the Environment of the Republic of Lithuania No. D1-79 of 27 January 2010 Environmental Protection Ministry, Ed. Valstybės žinios: Vilnius, Lithuania, 2010; Vol. No. 14-676.

Lithuanian State Forest Service, 2021. Lithuanian State Forest Stand Cadastral GIS Database. Ministry of Environment, State Forest Service, Vilnius, Lithuania.

Lithuanian State Forest Service. 2022. Lithuanian Statistical Yearbook of Forestry 2021; Ministry of Environment, State Forest Service. Vilnius, Lithuania.

Montenegro

According to the Law on Nature Protection of Montenegro (Official Gazette of Montenegro, N° 54/16) the protected natural areas are: strict nature reserve, national parks, special nature reserve, nature parks, monument of nature and regions of outstanding features. Strict nature reserves are particularly important among the mentioned categories. Strict nature reserve are areas with exceptional or representative ecosystems, unmodified or slightly modified overall nature. It is prohibited to perform actions or activities in the strict natural reserve. Strict nature reserves are predominantly intact or well-preserved forest ecosystems.

The forest ecosystem of Biogradska Gora National Park on Mt. Bjelasica developed through natural processes with no direct anthropogenic influences (Curovic *et al*. 2020). Their ecological value has been recognized since the end of the 19th century. The first official "protection" measures concerning forests of Biogradska Gora date back to 1878 and finaly Biogradska Gora was declared a national park in 1952. Forest ecosystems of Biogradska Gora are among the most significant and best-preserved old-growth forest relicts in the whole of south-eastern Europe (Cagliero *et al*. 2021).

Very important and well-preserved forest ecosystems are also found within the national parks: "Durmitor" and "Prokletije". In the National Parks, these forest complexes are within the different level of protection:

Protected area of category Ia which includes strictly protected areas set aside to protect biodiversity where use of space and impacts are strictly controlled and limited to ensure protection of the conservation values of the area.
Protected area of category Ib which includes protected areas that are large unmodified or slightly modified protected areas, which retained their natural character and influence, without permanent or significant human habitation, which are protected and managed so as to preserve their authentic state.
Protected area of category II which includes large natural areas set aside with the aim of protecting large-scale ecological processes, alongside with the complement of wild species of plants, animals and fungi and ecosystems that are characteristics of the area, which also provide a foundation for ecologically and culturally compatible spiritual, scientific, educational and recreational activities and visitor opportunities.

Cagliero, E., Morresi, D., Paradis, L., Curovic, M., Spalevic, V., Marchi, N., Meloni, F., Bentaleb, I., Motta, R., Garbarino, M., Lingua, E., Finsinger, W. (2021). Legacies of past human activities on one of the largest old-growth forests in the south-east European mountains. Veget Hist Archaeobot https://doi.org/10.1007/s00334-021-00862-x

Curovic, M., Spalevic, V., Sestras, P., Motta, R., Dan, C., Garbarino, M., Vitali, A., Urbinati, C. (2020). Structural and ecological characteristics of mixed broadleaved old-growth forest (Biogradska Gora - Montenegro). Turkish journal of Agriculture and Forestry, 44: 428-438. DOI:10.3906/tar-2003-103

Netherlands

The Dutch forest reserve network was established since 1983, and includes 60 strict reserves varying in size between 4 and 300 ha. The forest reserve network was established in response to the increased interest in natural processes in forest ecosystems. All reserves have been established in areas that were managed before, also in previous monoculture production forests. The reserves were chosen to represent specific forest and site types. Most of the reserves are administered by the National State Forest Service (Staatsbosbeheer). Monitoring, data management, and research are carried out mostly by Wageningen University and Research, and is described in detail in Broekmeyer (1999) and Bijlsma & Clerkx (2019), and can be accessed via http://www.bosreservaten.wur.nl/

Bijlsma, R.J. & A.P.P.M. Clerkx 2019. The Dutch forest reserve network; Documentation of monitoring design and databases. Wageningen Environmental Research, Report 2940. 36 pp. https://doi.org/10.18174/472664

Broekmeyer, M 1999. The Netherlands. In: J. Parviainen et al. (eds.)
Research in Forest Reserves and Natural Forests in European Countries. EFI Proceedings No. 16, p. 77-93.

Norway

In Norway, the main type of strictly protected forests (according to the definitions used in this paper) is the nature reserve, while a very limited number of national parks also have some forest within

a strictly protected zone. The nature reserves are typically small and scattered, with the 1692 forest nature reserves having an average size of 255,4 +/- 603,0 SD ha.

The list was compiled using a GIS procedure based on two officially available spatial databases:

- the protection regimes and forest area comes from N50 kartdata (N50 Kartdata - Kartkatalogen (geonorge.no) (as of 19.03.2022)
- to fit the stipulated requirements the forest areas were overlain by forest age data from SR16 Beta (SR16 beta - Nibio). (as of 19.03.2022).
- only the reserves that have >5 ha forest were retained.

Poland

In Poland, several types of protected areas exist, having different regime of protection. Strict protection that excludes timber production and other forms of forest management is obligatory in core parts of national parks (so-called strictly protected zone) and selected nature reserves. The general idea behind strict protection is to avoid interfering with natural ecosystem processes. However, there are three types of reserve protection at the national level, i.e. strict, active and landscape protection. Reserves under active protection are not managed for timber production. Although such sites may allow tree cutting operations, these activities result from clearly defined needs and the extent in which any cuts are carried out is recorded in official protection plans. Therefore, the active protection of a reserve essentially limits most activities, allowing management depending on the needs defined by the conservation objective of a reserve, e.g. certain restoration activities are possible. Areas under strict protection that excludes logging may, however, allow for felling of certain trees if, for example, required by safety reasons. However, the scale of such activities in areas under strict protection is legally limited and monitored. Landscape protection consists of managing agriculture, forestry or fisheries in a protected area in a way that takes into account the needs of the protected object. In general, human activities in national parks and nature reserves are limited to tourism, following strict regulations and allowed mostly in designed trails. The major type of tourism is hiking, with limited options for canoeing, climbing or skiing in few areas.

Currently, Poland's national parks account for 1% of the country's total area and nature reserves account for an additional 0.5%. The oldest national park and reserve were established in 1932 and 1938, respectively. The total forest area of Poland is 92647 km^2, which is approximately 30% of the country's total area (https://www.bdl.lasy.gov.pl/portal/Media/Default/Publikacje/GUS_lesnictwo_2022.pdf) and the total area of forest designated as Natura 2000 sites 32379 km^2 (https://www.bdl.lasy.gov.pl/portal/zestawienia). Vast majority of forest areas protected by national parks and nature reserves are designated as NATURA2000 areas.

Data on general area of national park and area that is under strict protection was taken from official sources (https://stat.gov.pl/obszary-tematyczne/srodowisko-energia/srodowisko/ochrona-srodowiska-w-2021-roku,12,4.html). There are 23 national parks in Poland, 21 of which have designated strictly protected zone. Data on nature reserves was taken from official database of Central Registry of Nature Conservation Forms (https://crfop.gdos.gov.pl), provided by General Directorate for Environmental Protection, the institution under the Ministry of Climate and Environment. The registry was accessed on 30.09.2022, providing information on 1506 nature reserves. As the database contains information on all nature reserves that has been legally established in the country, the data was filtered to include only nature reserves that meet the criteria set in the study. To this end each nature reserve has to protect forest habitat that is larger than 5 ha and do not allow for any form of forest management. If a nature reserve contains two or more ecosystems, then visual inspection of orthophoto map was conducted to estimate percentage of the area that is covered by forests. Some nature reserves are covered by forests, but the major aim of a reserve is to protect other resources, e.g. rock formations or historical monuments. In such objects more extensive cutting of trees is possible and, therefore, such objects were excluded from database. Reserves under landscape protection were also excluded. Finally, 1100 nature reserves were included in the database. The ultimately included reserves are typically small and scattered across the country, with median size of 44.7 ha (quartile range 19.8-109.4 ha, range 5.0-8581.6 ha) of the total area and 39.7 ha (quartile range 17.0-96.2 ha, range 5.0-8581.6 ha) of the forest area.

Romania

In Romania, the primary category of strictly protected forests (as per the definitions employed in this paper) is the scientific, natural reserves, and virgin forests (strictly protected), designated as the 1st degree of conservation status (T1) according to the national scale of conservation. Additionally, the national parks include strictly protected core zones (referred to as the 1st degree of conservation - T1). The list was assembled through a GIS procedure utilizing two spatial databases officially provided by the Ministry of Environment, Forests and Waters:
Strict reserves (1st degree of conservation status T1) Core zones of national parks (1st degree of conservation status T1)

Slovakia

In Slovakia, the primary category of strictly protected forests (as per the definitions employed in this paper) is the strict reserve, designated as the 5th degree of conservation status according to the national scale of conservation. Additionally, a restricted number of national parks include strictly protected core zones (referred to as A zones). The list was assembled through a GIS procedure utilizing two spatial databases officially provided by the Ministry of Environment and State Nature Conservation:

- Strict reserves (5th degree of conservation status)
- Core zones of national parks (A zones)

Slovenia

The national network of strict forest reserves in Slovenia consists of 164 reserves managed by the Slovenia forest service:
http://www.zgs.si/gozdovi_slovenije/o_gozdovih_slovenije/gozdni_rezervati/index.html.

Most reserves were established during the 1970s, except for a few old-growth reserves that were protected earlier in the 20th century. These reserves are the only forest ecosystems in the country where forest management is prohibited by law. The network was originally established to serve as a control for forest management and for research purposes, and less so for biodiversity conservation (Mlinsek et al., 1980). Most of the reserves are small (< 20 ha) and together make up less than 1% of the total forest area in the country; 16 forest reserves are classified as old-growth, which collectively represent 0.07% of the total forest area (Nagel et al., 2017). The only national park in Slovenia, Triglav National Park, contains a large core zone where regular wood extraction activities are not permitted (1st conservation zone). Forests cover approximately 22,422 ha in this core zone, about half of which is dominated by timberline forest vegetation (e.g. *Pinus mugo*). However, under the Act governing the National Park, sanitation and salvage logging are permitted under some circumstances. Therefore, forests in the core zone of the park were not included in this assessment. Finally, of the 164 reserves in the network, 126 are above 5 ha in size and are included in the data analyzed here.

Mlinsek, D., Accetto, M., Anko, B., Piskernik, M., Robic, D., Smolej, I., Zupancic, M., 1980. Forest Reserves in Slovenia. VTOZD za gozdarstvo, IGLG, Ljubljana.

Nagel, T.A., Firm, D., Pisek, R., Mihelic, T., Hladnik, D., de Groot, M., & Rozenbergar, D. 2017. Evaluating the influence of integrative forest management on old-growth habitat structures in a temperate forest region. *Biological Conservation* 216: 101–107.

Spain

Spain has a long history of deforestation and afforestation. In fact, most of the areas designated as protected areas were managed in the past so current conditions are still far from old-growth or primary forest. In some natural parks, zones are declared as strict/integral natural reserves or maximum protection zones, but without specification if harvest is allowed or not that make impossible the assignment to strict zone.

The Natural protection areas (NPAs) in Spain include several legal entities like National parks, natural reserves, natural monuments or natural parks depending on the autonomous region involved. These NPAs may or may not be within a Natura 2000 site that complicates the zonation. According to the latest annual forestry statistics review of Spain (MITECO, 2022) the forest area in NPAs (outside Natura 2000 sites) is 201 921 ha, which represents 1% of the total forest area in Spain.

Among NPAs figures the reserve zone in National parks and integral natural reserves are probably the strictest zones as defined in this paper. However, the information and public cartography of these sites is disperse and even lacking in some cases, making it difficult to compile a complete list of strictly protected forests areas.

We also included updated data on the amount of extensive and intensive forests in Spain for this study. Intensive management with fertilization, breeding, irrigation and clear felling is usually restricted to Eucalyptus, Radiata pine and Poplar plantations as well as other non-native tree species, for which the latest available annual forestry report indicates 1352532 ha, representing 7% or forest land in Spain (MITECO, 2022). We considered all other forests outside of strict protected areas and intensively managed areas as extensively managed forests. Some of these managed forests fall within Natura 2000 sites (3386432 ha). The silviculture in Natura 2000 sites aims to preserve and improve forest condition as well as to increase forest biodiversity. Consequently, silvicultural systems might follow continuous cover criteria. Although the management plans do not usually describe specific treatments, the practice is to apply extensive silviculture. Interestingly, in some cases where strict zone might be expected, (e.g. relict pinewoods) the total absence of intervention or strict protection would lead to a species composition change and the subsequent loss of the natural values the area holds. Consequently, silvicultural treatments to preserve the naturalness is allowed. The rest of the forest area outside the above figures represent several silvicultural regimes that might be placed, for example, between uneven-aged silviculture in silver fir stands in the Pyrenees to shelter-wood systems in Scots pine stands of the Iberian mountain range or clearcutting-with-reserves in *Pinus pinaster* stands of central Spain. The exact quantification of silvicultural systems should refer to management plans in a case by case basis, and it will be still incomplete as only 21.5% of forests have a formal management plan (MITECO, 2022)

MITECO-Ministry of Ecological Transition and Demographic Challenge, 2022. Anuario de Estadística Forestal – 2021.

Sweden

Statistics Sweden (SCB 2023) annually reports four categories of conservation areas. These include (i) formally protected areas, (ii) voluntarily set-aside forest stands in the context of forest certification systems, (iii) conservation management through tree retention and nature consideration patches, as well as (iv) other effective area-based conservation measures as a consequence of not allowing harvesting of unproductive forest areas (Table SWE-1).

Table SWE-1. Basic information about four groups of conservation instruments in Sweden (SCB 2023).

	(i.i) Formal protection; according to the Environmental Code		(i.ii) Formal protection; according to the Land Code	(ii) Voluntary set-asides	(iii) Nature considerations (§ 30, Forestry law)	(iv) Unproductive (wood production <1m3ha-1yr-1) (§ 13a, Forestry law)
Area and proportion of all forest land in 2022, totalling 28.1 million ha		(i.i and i.ii) 2,398 10^3 ha (8.9%)		(ii) 1,373 10^3 ha (4.9%)	(iii) 526 10^3 ha (1.9%)	(iv) 3,065 10^3 ha (11.0%)
Area and proportion of productive forest (wood production >1m3ha-1yr-1) in 2022, totalling 23.6 million ha		(i.i and i.ii) 1,349 10^3 ha (5.9%)		(ii) 1,373 10^3 ha (5.8%)	(iii) 526 10^3 ha (2.2%)	(iv) NA
Aim	**National park, nature reserve**: Conserve and develop nature of high value for plants, animals and people	**Biotope protection**: Conserve terrestrial or aquatic habitat for threatened species	**Conservation agreement**: Conserve and develop qualities for biodiversity	A complement to formal protection	Consideration to biodiversity conservation in managed forest	Wood harvest not recommended
Establishment	1909 and 1964, respectively	1998	1993	1995	1979	1979
Target size	Usually >20 ha	Usually <20 ha	Variable	>0.5 ha	< ca 0.5 ha	>0.1 ha
Duration	Permanent	Permanent	Variable	Unknown	Unknown	Permanent
Decision by	Parliament, Government, County, Municipality	Forest Agency, Municipality	Agreement between the State or Municipality and owner	Land owner	Parliament, Government, Forest Agency	Parliament, Government
Control	County	Forest Agency, Municipality	State	Forest certification	Forest Agency	Forest Agency
Monitoring	Georeferenced GIS polygons	Georeferenced GIS polygons	Georeferenced GIS polygons	GIS data and questionnaires	Random field sampling	National Forest Inventory

It is essential, however, to observe that areas and area proportions of the four types of denominations are not summed up in the official statistics. Reasons include that legal distinctions differ among these categories, how they may affect land use, as well as the methods used to estimate the areas. Altogether, this means that aggregating areas representing different categories without considering their effectiveness, may be severely misleading in relation to the objective to maintain viable populations of naturally occurring species by maintaining representative habitat networks (Angelstam et al. 2020).

With the focus on formally protected areas in this study we compiled polygons for the four categories: national park, nature reserve, biotope protection and conservation agreements (SCB 2023; see Table SWE-2). This were combined with the forest cover mask from the National Land Cover Data (Naturvårdsverket 2023), which includes both productive forests and low-productive forests. This corresponds to data presented for all formally protected forest presented in SCB (2023: Table 1.2.).

Table SWE-2. Categories of formally protected areas (sub-categories checked with Gunilla Olsson at SEPA).

Main group of formal protection	Sub-category
National park	National park
Nature reserve	Nature reserve
	Natura 2000 forest habitat
	Swedish Fortifications Agency
	Nature reserve in progress
Biotope protection	Forest biotope protection
	Forest biotope protection in progress
Conservation agreement	Conservation agreement with Swedish EPA and Forest Agency
	Sveaskog Ekopark and white-backed woodpecker area

Especially boreal forest landscapes, which dominate in central and northern Sweden (see table and figure 3), are mixtures of forests and mires. As protected areas encompass both these land covers, the forest cover is thus commonly divided into several GIS polygons.

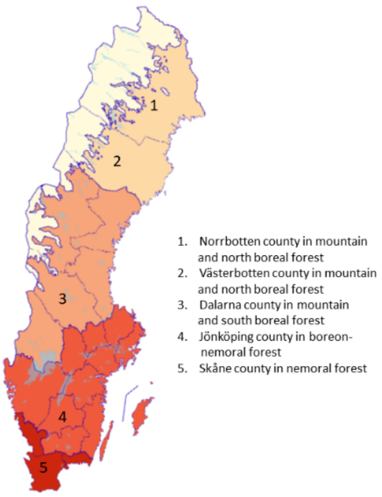

Table/Figure SWE 3. Examples of Swedish counties with different forest and mire proportions, mire/forest ratios, formally protected forest, and proportion all formally protected areas in Sweden.

County	Forest proportion	Mire proportion	Ratio mire/forest	Protected forest (ha)	Proportion of all protected areas
1. Norrbotten	0.41	0.19	0.46	1 146 900	0.48
2. Västerbotten	0.58	0.17	0.29	417 400	0.17
3. Dalarna	0.71	0.15	0.22	165 500	0.07
4. Jönköping	0.69	0.06	0.09	14 000	0.01
5 Skåne	0.39	0.02	0.04	20 100	0.01

Figure SWE4. Maps of Muddus NP in Norrbotten county (top) and Björnlandet NP in Västerbotten county (bottom) showing the mixture of forests and mires.

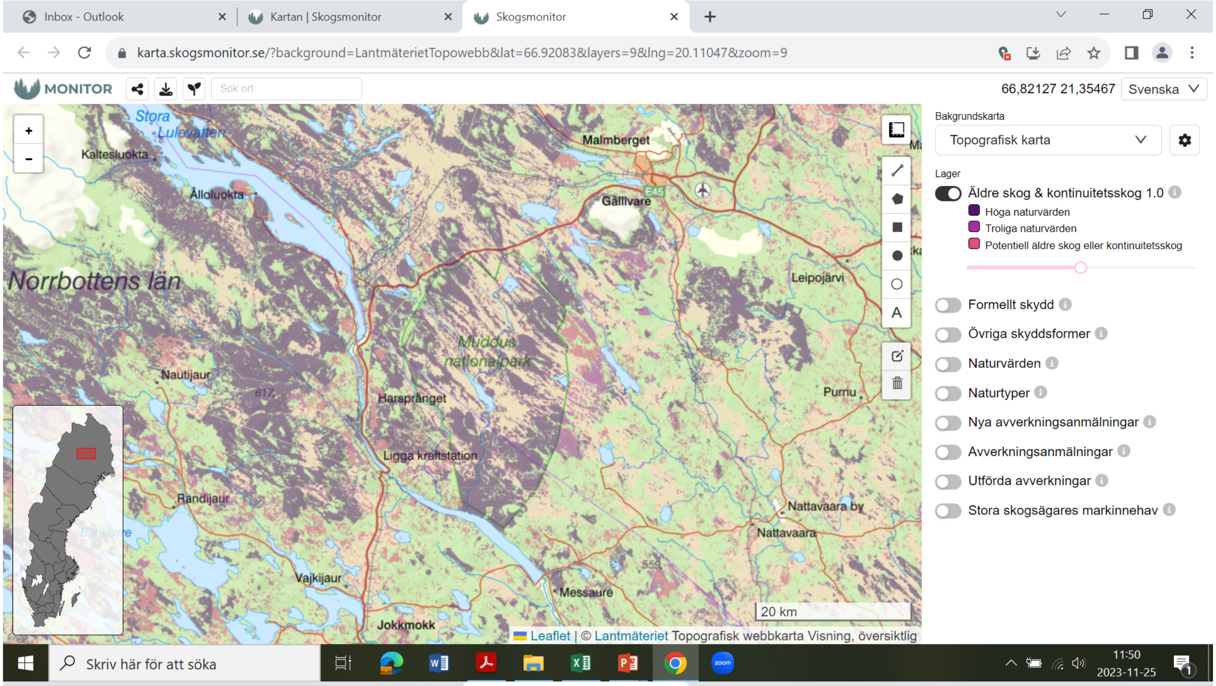
Inbox - Outlook
Kartan | Skogsmonitor
Skogsmonitor
karta.skogsmonitor.se/?background=LantmäterietTopowebb&lat=66.92083&layers=9&lng=20.11047&zoom=9
MONITOR
Sök ort
66,82127 21,35467
Svenska
Stora Luleälven
Kaltesluokta
Malmberget
Gällivare
Ålloluokta
Norrbottens län
Leipojärvi
Muddus nationalpark
Nautijaur
Harsprånget
Purnu
Ligga kraftstation
Randijaur
Nattavaara by
Nattavaara
Vajkijaur
Messaure
Jokkmokk
20 km
Leaflet | © Lantmäteriet Topografisk webbkarta Visning, översiktlig
Bakgrundskarta
Topografisk karta
Lager
Äldre skog & kontinuitetsskog 1.0
Höga naturvärden
Troliga naturvärden
Potentiell äldre skog eller kontinuitetsskog
Formellt skydd
Övriga skyddsformer
Naturvärden
Naturtyper
Nya avverkningsanmälningar
Avverkningsanmälningar
Utförda avverkningar
Stora skogsägares markinnehav
Skriv här för att söka
11:50
2023-11-25

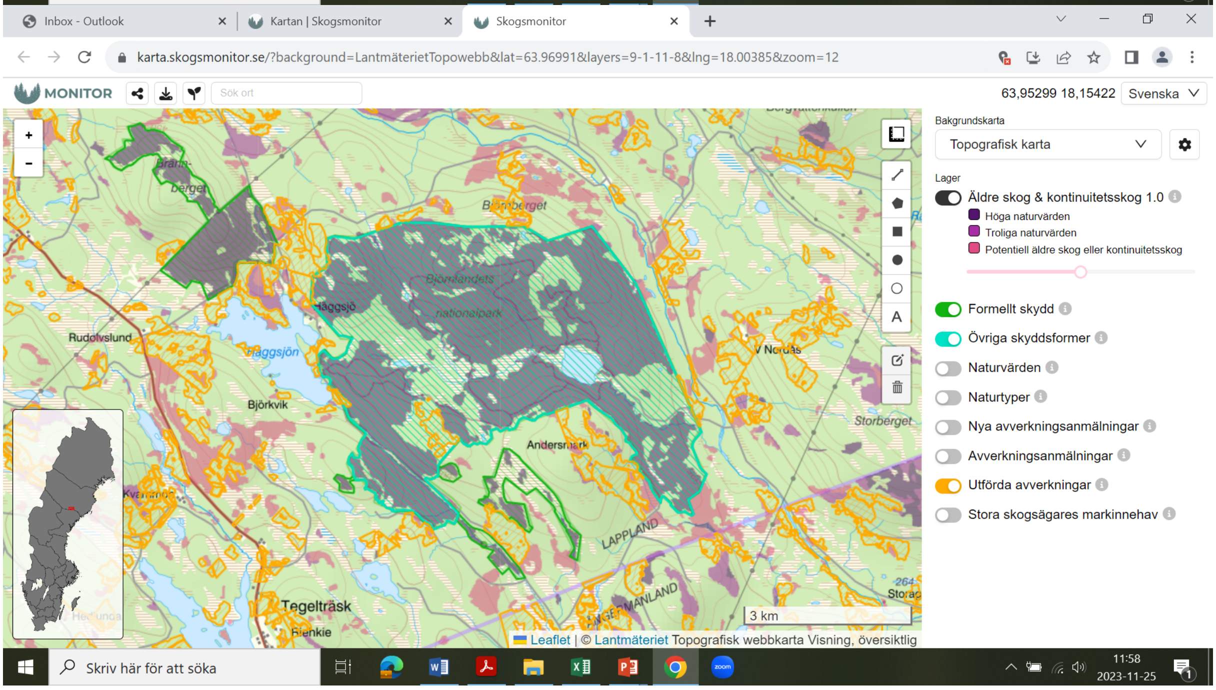
Inbox - Outlook
Kartan | Skogsmonitor
Skogsmonitor
karta.skogsmonitor.se/?background=LantmäterietTopowebb&lat=63.96991&layers=9-1-11-8&lng=18.00385&zoom=12
MONITOR
Sök ort
63,95299 18,15422
Svenska
Björnberget
Björnlandets nationalpark
Häggsjö
Rudolvslund
Häggsjön
Björkvik
Andersmark
Storberget
LAPPLAND
ÅNGERMANLAND
Tegelträsk
3 km
Leaflet | © Lantmäteriet Topografisk webbkarta Visning, översiktlig
Bakgrundskarta
Topografisk karta
Lager
Äldre skog & kontinuitetsskog 1.0
Höga naturvärden
Troliga naturvärden
Potentiell äldre skog eller kontinuitetsskog
Formellt skydd
Övriga skyddsformer
Naturvärden
Naturtyper
Nya avverkningsanmälningar
Avverkningsanmälningar
Utförda avverkningar
Stora skogsägares markinnehav
Skriv här för att söka
11:58
2023-11-25

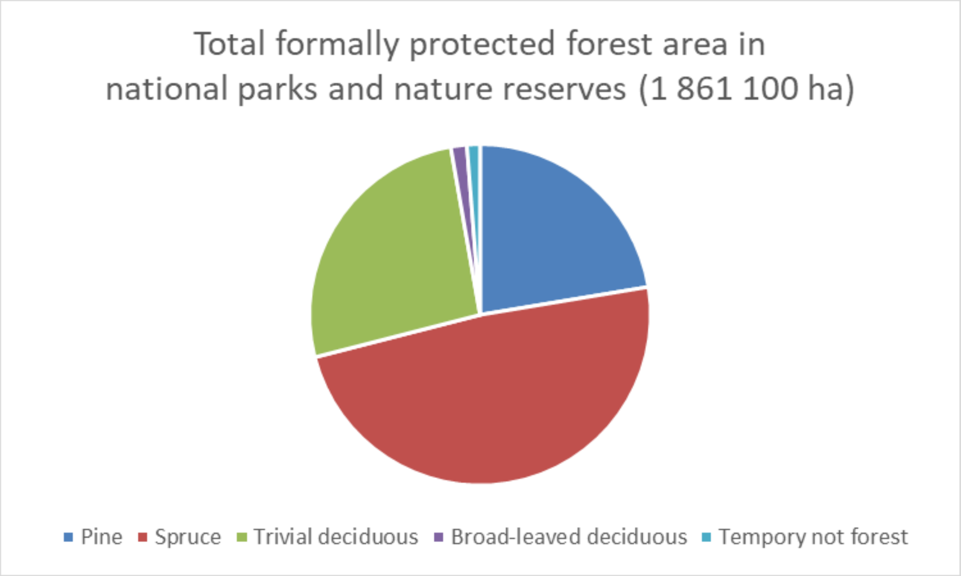

References

Angelstam, P., Bush, T., Manton, M. 2023. Challenges and Solutions for Forest Biodiversity Conservation in Sweden: Assessment of Policy, Implementation Outputs, and Consequences. Land 12: 1098. https://doi.org/10.3390/land12051098

Naturvårdsverket. 2023. Nationella marktäckedata 2018 basskikt version 2.3. Naturvårdsverket, Stockholm. Available at: https://geodata.naturvardsverket.se/nedladdning/marktacke/NMD2018/NMD_Produktbeskrivning_NMD2018Basskikt.pdf

SCB (Statistiska centralbyrån). 2023. Formellt skyddad skogsmark, frivilliga avsättningar, hänsynsytor samt improduktiv skogsmark (Formally protected forestland, voluntary set-asides, retention patches and unproductive forestland). MI 41 2022A02 (Available as URN:NBN:SE:SCB-2023: MI41BR2302_pdf at https://www.scb.se/hitta-statistik/statistik-efter-amne/miljo/markanvandning/formellt-skyddad-skogsmark-frivilliga-avsattningar-hansynsytor-samt-improduktiv-skogsmark/pong/publikationer/formellt-skyddad-skogsmark-frivilliga-avsattningar-hansynsytor-samt-improduktiv-skogsmark-2022/)

Switzerland

Brief description of the data entered into the database:

The data from Switzerland establish the state of the forest reserves in Switzerland at the end of 2018. The data were delivered by the cantons for the national statistics. These statistics are described in the following report:
Eisenhut A., Mattli J., Stöckli B. (2019): Waldreservate in der Schweiz. Bericht über den Stand Ende 2018. Impuls AG, Thun. 18 S.

Data preparation

1. Combine forest reserve data from the cantons and from Pro Natura (Swiss NGO, which also owns forest reserves).
2. Merge polygons per forest reserve id regarding the protection status (MCPFE 1.1. to 1.3).
3. Overlay with the forest layer. For this, we used the Layer Swisstopo SwissTLM3D 2019 (Wald).
4. Extract the surface for each forest reserve id regarding the protection status.

Description of the strict forest network

According to the Forest law, cantons are responsible for setting aside forest reserves. The federal government provides the strategic and technical basis and supports the cantonal reserve programs with subsidies. Forest reserves are permanently protected areas that are contractually secured for a limited period of time between the canton and forest owners (usually for 25 years for special forest reserves, or 50 years, or less frequently 99 years for natural forest reserves).

Forest reserves protect the forest as a natural ecosystem and enable the conservation of biodiversity. There are 2 kinds of forests reserves: (1) Natural forest reserves or strict forest reserves: no management is made at all so that the forest can develop without human interventions (MCPFE 1.1: no intervention; 1.2: minimum intervention); (2) Special forest reserves: targeted measures are made to promote endangered species. These include, above all, heliophilous species (MCPFE 1.3: conservation through active management). Special forest reserves were not included in this study.

If both types of reserve are combined, they are referred to as complex reserves.

From:
https://www.bafu.admin.ch/bafu/de/home/themen/biodiversitaet/fachinformationen/oekologische-infrastruktur/waldreservate.html

Turkey

All protected areas containing forests and trees in Türkiye are under public ownership. These areas are managed by the General Directorate of Nature Conservation and National Parks and the General Directorate of Forestry under the Ministry of Agriculture and Forestry according to Turkish law. Protected areas in Türkiye are classified according to their characteristics as national parks, nature reserves, nature parks, natural monuments, wetlands, conservation forests, gene conservation forests, seed stands and wildlife protection areas. Wood production and hunting are not allowed in these areas. Recreational activities (camping, accommodation, picnic, trekking) can be carried out in a limited and controlled way in certain areas.

Important natural sites in Türkiye are protected by many different protection statuses. In some cases, a single area may be given more than one protection status. While the majority of these areas are divided according to the definitions made according to Turkish legislation, some of them are based on international agreements (i.e. IUCN, Ramsar).

According to Turkish legislation, "National Park" is defined as a part of nature with national and international rare cultural and natural resource values in terms of science and aesthetics, as well as recreation, protection and tourism areas. The parts of nature that have wildlife and vegetation characteristics and are suitable for recreation and leisure of people within the framework of landscape unity constitute "Natural Parks". "Natural Monuments" are defined as the parts of nature that have scientific value with the features revealed by nature and natural events and are protected within the framework of national park criteria. "Nature Reserve" refers to the parts of nature that contain rare, endangered or nearly disappearing ecosystems, varieties and distinguished precedents created by natural events, which are important for science and education, and which must be preserved without exception, and which are reserved for the sole purpose of being used for science and education purposes. "Wildlife Conservation Area" means the areas where the habitats that have wildlife values and need to be protected are absolutely protected together with plant and animal species and their continuity is ensured. "Wetland" is defined as artificial or natural, permanent or seasonal, fresh or salty, stagnant or flowing waters, marshes and sea waters not exceeding 6 metres in depth at low tide (National Parks Law, Law no. 2873, 1983).

"Conservation Forests" are the forests in places subject to the danger of being destroyed by landslides and rains, and the state forests that protect the air of residential areas, roads and railways against dust and sandstorms, prevent the filling of river beds or prevent the filling of river beds, or are deemed necessary for the defence of the country and are covered with state forests, shrubs or bushes that are set aside for these reasons. "Gene Conservation Forests" are natural stands managed to preserve

the genetic diversity of a species in its natural environment (in-situ). "Seed Stands" are areas whose coordinates are determined and managed by the Ministry of Agriculture and Forestry in order to produce seeds of forest tree and shrub species (www.tarimorman.gov.tr, 2023).

The list and areas of strict reserves from protected areas in Türkiye and the forest areas they cover were obtained from the data published on the official websites of the General Directorate of Nature Conservation and National Parks and the General Directorate of Forestry under the Ministry of Agriculture and Forestry.
